# Pediatric obstructive sleep apnea diagnosis: leveraging machine learning with linear discriminant analysis

**DOI:** 10.3389/fped.2024.1328209

**Published:** 2024-02-14

**Authors:** Han Qin, Liping Zhang, Xiaodan Li, Zhifei Xu, Jie Zhang, Shengcai Wang, Li Zheng, Tingting Ji, Lin Mei, Yaru Kong, Xinbei Jia, Yi Lei, Yuwei Qi, Jie Ji, Xin Ni, Qing Wang, Jun Tai

**Affiliations:** ^1^ Department of Child Health Care, Children’s Hospital Capital Institute of Pediatrics, Chinese Academy of Medical Sciences & Peking Union Medical College, Capital Institute of Pediatrics, Beijing, China; ^2^ Pharmacovigilance Research Center for Information Technology and Data Science, Cross-strait Tsinghua Research Institute, Xiamen, China; ^3^ Department of Otolaryngology, Head and Neck Surgery, Beijing Children's Hospital, Capital Medical University, National Center for Children's Health, Beijing, China; ^4^ Respiratory Department, Beijing Children's Hospital, Capital Medical University, National Center for Children's Health, Beijing, China; ^5^ Faculty of Information Technology, Beijing University of Technology, Beijing, China; ^6^ Department of Otolaryngology, Head and Neck Surgery, Children’s Hospital Capital Institute of Pediatrics, Beijing, China; ^7^ Department of Automation, Tsinghua University, Beijing, China

**Keywords:** obstructive sleep apnea, machine learning, artificial intelligence, computer-aided diagnosis, children

## Abstract

**Objective:**

The objective of this study was to investigate the effectiveness of a machine learning algorithm in diagnosing OSA in children based on clinical features that can be obtained in nonnocturnal and nonmedical environments.

**Patients and methods:**

This study was conducted at Beijing Children's Hospital from April 2018 to October 2019. The participants in this study were 2464 children aged 3–18 suspected of having OSA who underwent clinical data collection and polysomnography(PSG). Participants’ data were randomly divided into a training set and a testing set at a ratio of 8:2. The elastic net algorithm was used for feature selection to simplify the model. Stratified 10-fold cross-validation was repeated five times to ensure the robustness of the results.

**Results:**

Feature selection using Elastic Net resulted in 47 features for AHI ≥5 and 31 features for AHI ≥10 being retained. The machine learning model using these selected features achieved an average AUC of 0.73 for AHI ≥5 and 0.78 for AHI ≥10 when tested externally, outperforming models based on PSG questionnaire features. Linear Discriminant Analysis using the selected features identified OSA with a sensitivity of 44% and specificity of 90%, providing a feasible clinical alternative to PSG for stratifying OSA severity.

**Conclusions:**

This study shows that a machine learning model based on children's clinical features effectively identifies OSA in children. Establishing a machine learning screening model based on the clinical features of the target population may be a feasible clinical alternative to nocturnal OSA sleep diagnosis.

## 1 Introduction

Obstructive sleep apnea (OSA) is a severe type of sleep-disordered breathing (SDB) characterized by recurrent events, including instances of partial or complete obstruction of the upper airway and disruptions in normal oxygenation, ventilation, and sleep patterns (1). In children, the estimated prevalence of OSA is 1%–5% (2). However, it is notably higher, ranging from 33% to 61%, among youths who are diagnosed with obesity (3). Untreated OSA has been linked to disturbances in both the cardiovascular and metabolic systems, as well as neurocognitive and behavioral dysfunction (4). Additionally, it is associated with hypertension (5), diminished school performance (6), growth failure (7), and a decline in overall quality of life (8).

Polysomnography (PSG) is the gold standard for diagnosing and characterizing OSA (2). However, it has drawbacks, such as being costly, time-consuming, and requiring specialized facilities and staff. Data collection of PSG requires an overnight hospital stay in a specially equipped sleeping suite involving more than 15 measurement channels (9). Based on these limitations, previous research indicates that over 80% of individuals with OSA are estimated to remain undiagnosed (10), and only 5%–10% of children receive PSG before adenotonsillectomy, leading to potential overuse of surgeries and postoperative risks (11). There is a view that developing a practical and affordable OSA diagnostic model would greatly benefit children in areas with limited sleep laboratory facilities (12).

Machine Learning (ML) methods represent an evolving approach capable of simultaneously and autonomously processing substantial volumes of data, continually refining their classification performance through previous experiences (13). Particularly adept at discerning patterns within data featuring numerous variables (14), ML methods harness extensive clinical datasets to craft practical diagnostic tools. In recent years, promising results have been reported in studies involving ML methods that facilitate OSA diagnosis using children's clinical features to develop diagnostic tools (15, 16), even to classify sleep stages in children with OSA (17). Most OSA diagnosis studies using ML methods rely on nocturnal biological signals such as Electrocardiogram (ECG), Electroencephalogram (EEG), Oxygen saturation (SpO2), and airflow signals to build diagnostic models (18). Compared with PSG, this approach dramatically reduces the number of biomarkers collected but still requires specialized electronic equipment for data collection.

Using clinical features and questionnaires is a cost-effective approach for identifying children with OSA, circumventing the need for specialized laboratory and sleep monitoring equipment (19–23). Notably, a study has enhanced the efficacy of OSA screening by using Selected Features and optimizing existing ones, demonstrating improved performance after eliminating redundancy features (24). However, these studies do not use cross-validation or test sets to verify classification performance. Given these considerations, our hypothesis posits that a machine learning approach, integrating multiple clinical variables, can more effectively identify pediatric OSA than standalone questionnaires. Consequently, this study pursues dual objectives: (1) Construction of a machine learning model solely relying on non-invasive clinical features for pediatric OSA diagnosis, and (2) Assessment and comparison of its performance against models derived from established sleep questionnaires.

## 2 Materials and methods

### 2.1 Dataset and inclusion and exclusion criteria

The study received approval from the Ethics Committee of Beijing Children's Hospital, Capital Medical University [(2022)-E-111-R]. Data collection occurred between April 2018 and October 2019 and involved children aged 3–18 years suspected of having OSA. Written informed consent was obtained from the parent or guardian of each participating child. A specialized physician measured the children's height, weight, neck, waist, and hip circumference before PSG. Additionally, the parents or guardians filled out an information collection form to provide the necessary features for the study.

Exclusion criteria included missing demographic information, presence of other disease-causing disordered breathing (e.g., neuromuscular diseases, craniofacial dysplasia, Down syndrome), chronic lung diseases, previous adenoidectomy and/or tonsillectomy, respiratory infection within the last three weeks, total sleep time less than 5 h, and non-completion of the questionnaire. The study adhered to the reporting guidelines of the Transparent Reporting of a multivariable prediction model for Individual Prognosis or Diagnosis (TRIPOD) statement (25).

### 2.2 Clinical feature data collection

The features used in this study were collected from no wearable devices or other overnight biomarker recordings. (1) Demographic and anthropometric data, including age, sex, neck circumference, abdominal circumference, hip circumference, and Body mass index (BMI), were collected. Overweight status was assessed according to the BMI standard for children aged 2–18 in China (26). The ratios of neck circumference to height, waist circumference to height, hip circumference to height, and waist to hip ratio were also calculated. (2) Children's symptoms and living habits were determined based on existing questionnaires such as the Pediatric Sleep Questionnaire (PSQ) (27), the Obstructive Sleep Apnea-18 (OSA-18) (28), and the Hong Kong Children's Sleep Questionnaire (HK-CSQ) (29). Some questions were refined to provide more information. In addition, we also collected information about family members, including the prevalence of snoring, OSA, and smoking, as well as the educational level of the parents. A total of 102 features have been collected, and a list of these features is shown in Supplementary Material 1.

Specialized doctors collected the above data using a questionnaire. The questionnaire was designed to gather clinical features, not as a screening test, and was formulated in simple language consistent with the local culture. Any confusing questions were clarified by a doctor.

### 2.3 Polysomnography

All patients underwent standard nocturnal PSG at Beijing Children's Hospital of Capital Medical University. The Alice 5 PSG device (Philips, Amsterdam, The Netherlands) was used for data collection. The data were manually scored according to the American Academy of Sleep Medicine (AASM) 2012 scoring criteria (30). Obstructive apnea was defined as a greater than 90% reduction in oronasal flow for at least two respiratory cycles, accompanied by respiratory efforts throughout the event. Hypopnea is defined as a reduction in airflow of at least ≥30%, accompanied by event-related arousal or oxygen desaturations of >3%, which persists for a minimum of two respiratory cycles. The apnea hypopnea index (AHI) was defined as the mean number of obstructive apnea and hypopnea events per hour during sleep. In this study, AHI ≥5 and AHI ≥10 were used as grouping criteria. The statistical differences between these groups are shown in Supplementary Material 1.

### 2.4 Data preprocessing

#### 2.4.1 Raw datasets

The study included 2,464 patients (1,665 boys, 799 girls). Each participant in the raw dataset had 102 features and one label column. The label values were encoded as 1 for the positive class and 0 for the negative class. Python 3.6 and the open-source Python automated machine learning library, PyCaret 2.3.10, were used for data preprocessing, modeling, evaluation, statistical analysis, and feature analysis33 (31).

#### 2.4.2 Raw data preprocessing

**Figure 1:**
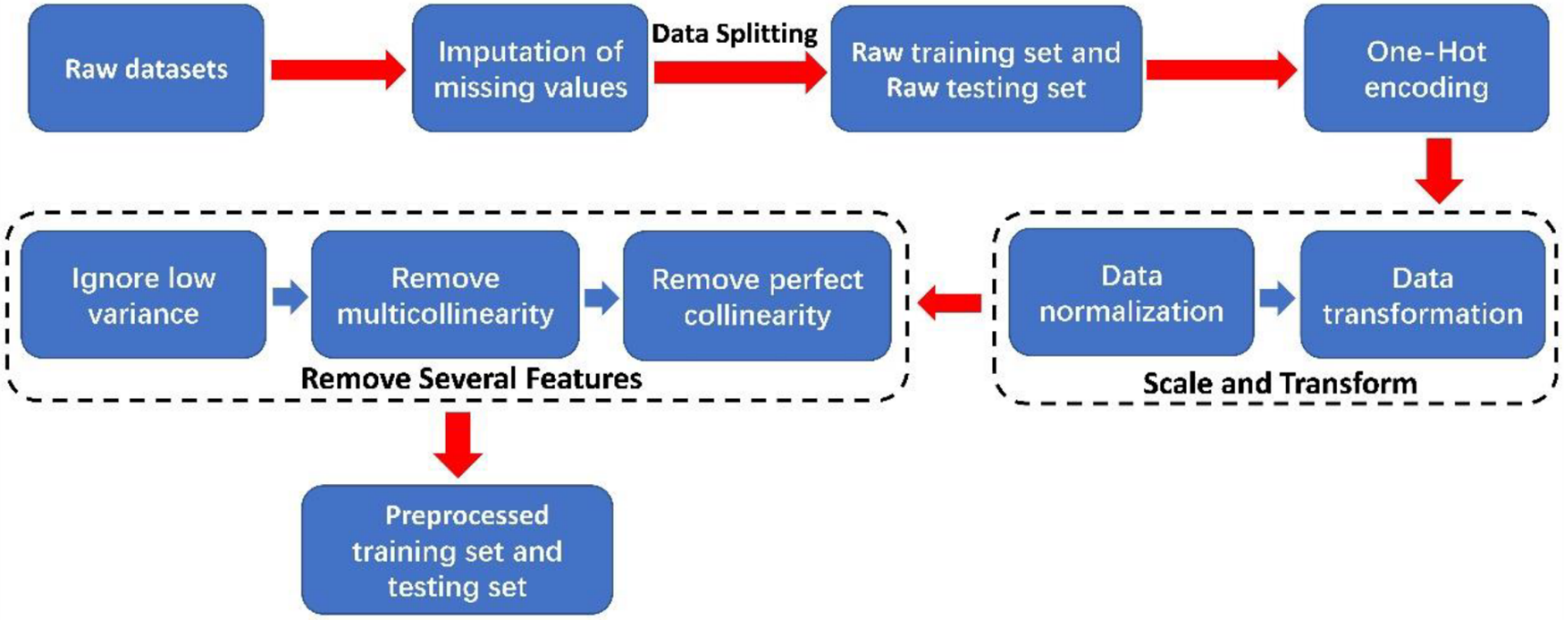
The block diagram of raw dataset preprocessing.

In this paper, the process of preprocessing the raw dataset is shown in Figure 1. Initially, missing values in categorical features were imputed with “NaN”. The dataset was then randomly split into a training set (1,971 samples) and a testing set (493 samples) with an 8:2 ratio. Afterward, one-hot encoding was applied to categorical features. Subsequently, we used the “z-score” normalization methods to perform data normalization and employed the “Yeo-Johnson” transformation methods to perform data transformation on continuous features (32). The “z-score” normalization method is used to find out the mean and standard deviation of the sample feature “x” and then use “(x-mean)/std” to replace the original feature value. The mean of each feature after “z-score” standardization is 0, and the standard deviation is 1. The “Yeo-Johnson” transformation methods bring the shape of the probability density function of the features closer to a normal distribution.

### 2.5 Machine learning methods

Instead of complex models with limited interpretability, six common ML methods were used for data modeling and evaluated for pediatric OSA binary classification performance using actual clinical data during cross-validation (33) as reported in previous studies (34–36). ML methods included logistic regression (LR) (37), linear discriminant analysis (LDA) (38), radial basis function kernel support vector machine (RBF-SVM) (39–41), CatBoost (42), AdaBoost (43), and random forest classifiers (RF) (44).

Several common metrics were implemented for the performance evaluation of each ML algorithm on the training and testing sets, including accuracy, balanced accuracy (BA), the area under the receiver operating characteristic curve (AUC) (45), positive predictive value (PPV), negative predictive value (NPV), sensitivity and specificity. Accuracy refers to the proportion of correctly identified samples in all samples. Balanced accuracy is a corrected measure of accuracy used for comparing datasets with imbalances in sample size. Higher AUC values indicate better classification performance of the model. The probability threshold was set to 0.5.

### 2.6 The procedure of data modeling

**Figure 2:**
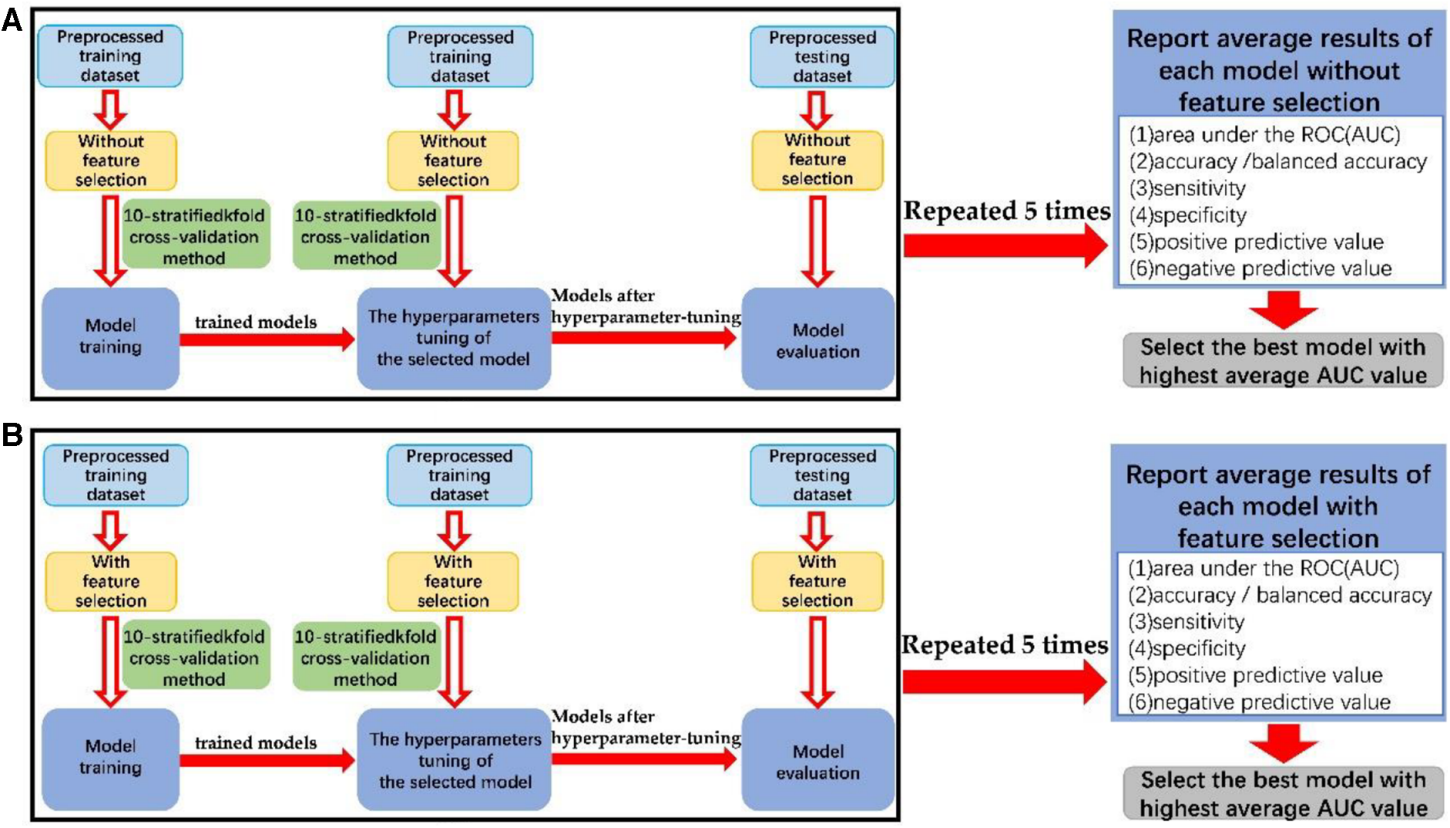
The block diagram of the process of data modeling (**A**) without feature selection and (**B**) with feature selection.

The data modeling procedure, as shown in Figure 2, was divided into three stages: model training, hyperparameter tuning of the selected model, and model evaluation. Hyperparameter tuning was performed using a random grid search, and the highest AUC value was used as the target. Five random seed values were utilized for data splitting during preprocessing to reduce bias (46). Six metrics were obtained from each hyperparameter-tuned ML model on the testing datasets. The average AUC value was calculated from five repetitions of the data modeling process, and other evaluation metrics were obtained similarly.

The preprocessed training datasets underwent model training and optimization using 10-stratified k-fold cross-validation. Overfitting was assessed by comparing results between training and testing datasets, and the selected hyperparameters were evaluated by comparing the trained model with the hyperparameter-tuned model. More reliable estimates of model performance were obtained through 10-stratified k-fold cross-validation in each repetition, ensuring consistent class distribution in each fold. The hyperparameters tuned through cross-validation of each machine learning algorithm is shown in Supplementary Material 5.

In addition, the best model was then trained and tested on preprocessed datasets that included 22 features of the PSQ questionnaire to predict the severity of OSA.

### 2.7 The procedure of feature selection

To select predictive features for OSA samples and create a concise model, we employed the Elastic net method as the feature selection algorithm (47). This method applies L1 and L2 penalties during training to shrink coefficients of unimportant features to zero. The Elastic net was implemented on all preprocessed datasets with two optimal parameters (alpha and L1_ratio) using the ElasticNet and ElasticNetCV functions (48). Features with coefficients having an absolute value greater than zero were retained, while those with a coefficient of zero were eliminated.

The feature selection procedure and optimal alpha and L1_ratio values are demonstrated in Supplementary Material 2. Finally, we performed a paired t test to compare the predictive performance of the best model based on PSQ and our selected feature set when an AHI greater than 5 and 10 events/h were the classification criteria. *P* < 0.05 was considered significant.

## 3 Results

After feature selection, 31 features were retained for a binary classification threshold of AHI ≥10 events/h and 47 features for AHI ≥5 events/h. The selected features are listed in Supplementary Material 3, and the details of the feature coefficients’ absolute values are reported in Supplementary Material 4. Twenty-seven features are simultaneously selected by machine learning algorithms for diagnostic tasks with AHI thresholds of 5 and 10. Among them, Sex has been proven to be a risk factor for OSA in children (49). Five features associated with Children's performance during bedtime had the highest feature importance values, indicating that these five features best-discriminated patients with vs. without moderate to severe pediatric OSA. The five features are A2. Snore more than half the time; A3. Always snore; A4. Snore loudly; A6. Have trouble breathing or struggle to breathe; A7. Stop breathing during the night; Q4. Mouth breathing during sleep. Based on the result of the feature coefficients’ absolute values, four features associated with Children's body measurement data and one feature associated with characteristics of family members were also used in the final model, e.g., Neck circumference, Hip circumference, Neck/height_ratio, Waist/hip_ratio, the educational level of the mother.

We compared the performance of six machine learning models for predicting OSA. Table 1 summarizes their average performance, obtained by repeating the data modeling process five times on the test datasets with all features. The performance was evaluated using six metrics, expressed as mean ± standard deviation. The results showed that the LDA classifier outperformed other models with the highest average AUC value of 0.73 and a mean accuracy rate of 68% when using an AHI of 5 events/h as the binary classification threshold. For the AHI of 10 events/h threshold, the LDA classifier remained the best model with the highest average AUC value of 0.77 and a mean balanced accuracy rate of 66%.

**Table 1:** Performance of each machine learning model using all features on test dataset (mean ± SD).

		Catboost	Adaboost	LDA	RF	RBF-SVM	LR
The machine learning models performance of AHI ≥5	**Accuracy**	0.68 ± 0.02	0.68 ± 0.02	0.68 ± 0.02	0.66 ± 0.03	0.66 ± 0.03	0.65 ± 0.02
	**AUC**	0.72 ± 0.01	0.72 ± 0.01	**0.73 **± **0.01**	0.72 ± 0.02	0.72 ± 0.02	0.71 ± 0.02
	**Sensitivity**	0.45 ± 0.05	0.46 ± 0.06	0.55 ± 0.05	0.51 ± 0.20	0.62 ± 0.05	0.58 ± 0.07
	**Specificity**	0.83 ± 0.03	0.83 ± 0.04	0.76 ± 0.02	0.77 ± 0.13	0.70 ± 0.04	0.70 ± 0.08
	**PPV**	0.65 ± 0.04	0.65 ± 0.04	0.62 ± 0.04	0.64 ± 0.11	0.58 ± 0.03	0.57 ± 0.05
	**NPV**	0.69 ± 0.03	0.69 ± 0.04	0.71 ± 0.03	0.70 ± 0.05	0.73 ± 0.03	0.71 ± 0.04
The machine learning models performance of AHI ≥10	**Balanced Accuracy**	0.59 ± 0.03	0.59 ± 0.02	0.66 ± 0.03	0.67 ± 0.04	0.66 ± 0.03	0.63 ± 0.02
	**AUC**	0.76 ± 0.02	0.75 ± 0.02	**0.77 **± **0.03**	0.75 ± 0.03	0.75 ± 0.03	0.74 ± 0.03
	**Sensitivity**	0.21 ± 0.06	0.21 ± 0.06	0.44 ± 0.06	0.53 ± 0.15	0.53 ± 0.14	0.37 ± 0.12
	**Specificity**	0.97 ± 0.01	0.97 ± 0.01	0.88 ± 0.01	0.80 ± 0.10	0.79 ± 0.09	0.89 ± 0.09
	**PPV**	0.69 ± 0.07	0.64 ± 0.05	0.52 ± 0.05	0.46 ± 0.10	0.45 ± 0.10	0.53 ± 0.10
	**NPV**	0.81 ± 0.02	0.81 ± 0.02	0.84 ± 0.02	0.86 ± 0.03	0.86 ± 0.01	0.83 ± 0.02

AHI, apnea hypopnea index; AUC, area under the receiver operating characteristic curve; PPV, positive predictive value; NPV, negative predictive value.

The AUC values with the best performance are provided in bold.

The performance of the selected features obtained by repeating the data modeling process five times on the test datasets is shown in Table 2. The LDA classifier achieved the highest average AUC value of 0.73 and a mean accuracy rate of 68% for the AHI ≥5 group. Additionally, it had the highest average AUC value of 0.78 and a mean balanced accuracy rate of 67% for the AHI ≥10 group.

**Table 2:** Performance of each machine learning model using selected features on test dataset (mean ± SD).

		Catboost	Adaboost	LDA	RF	RBF-SVM	LR
The machine learning models performance of AHI ≥5	**Accuracy**	0.68 ± 0.02	0.68 ± 0.03	0.68 ± 0.01	0.67 ± 0.02	0.67 ± 0.02	0.68 ± 0.02
	**AUC**	0.72 ± 0.01	0.71 ± 0.02	**0.73 **± **0.02**	0.73 ± 0.01	0.72 ± 0.03	0.72 ± 0.02
	**Sensitivity**	0.46 ± 0.04	0.47 ± 0.05	0.58 ± 0.03	0.56 ± 0.11	0.63 ± 0.04	0.60 ± 0.10
	**Specificity**	0.83 ± 0.04	0.82 ± 0.04	0.76 ± 0.02	0.74 ± 0.10	0.69 ± 0.04	0.73 ± 0.08
	**PPV**	0.65 ± 0.04	0.65 ± 0.04	0.62 ± 0.03	0.61 ± 0.07	0.58 ± 0.02	0.61 ± 0.05
	**NPV**	0.69 ± 0.03	0.69 ± 0.04	0.72 ± 0.03	0.72 ± 0.04	0.73 ± 0.03	0.73 ± 0.04
The machine learning models performance of AHI ≥10	**Balanced Accuracy**	0.59 ± 0.02	0.59 ± 0.02	0.67 ± 0.01	0.66 ± 0.05	0.70 ± 0.02	0.62 ± 0.01
	**AUC**	0.77 ± 0.01	0.76 ± 0.02	**0.78 **± **0.02**	0.78 ± 0.02	0.77 ± 0.02	0.77 ± 0.03
	**Sensitivity**	0.21 ± 0.05	0.23 ± 0.04	0.44 ± 0.03	0.44 ± 0.17	0.66 ± 0.02	0.31 ± 0.03
	**Specificity**	0.97 ± 0.01	0.96 ± 0.01	0.90 ± 0.02	0.87 ± 0.07	0.74 ± 0.02	0.94 ± 0.01
	**PPV**	0.66 ± 0.05	0.61 ± 0.05	0.57 ± 0.06	0.54 ± 0.12	0.42 ± 0.04	0.61 ± 0.06
	**NPV**	0.81 ± 0.02	0.81 ± 0.02	0.85 ± 0.01	0.85 ± 0.03	0.88 ± 0.01	0.82 ± 0.02

AHI, apnea hypopnea index; AUC, area under the receiver operating characteristic curve; PPV, positive predictive value; NPV, negative predictive value.

The AUC values with the best performance are provided in bold.

Furthermore, we compared the diagnostic ability of feature selection with the PSQ questionnaire using the LDA algorithm. The results, presented in Table 3, demonstrate that the LDA model of feature selection had a higher AUC value, sensitivity, and NPV than PSQ for classifying both the AHI ≥5 and AHI ≥10 groups (*P* < 0.05). These findings demonstrate that our selected features, when analyzed using the LDA algorithm, offer superior diagnostic accuracy and classification ability compared to the traditional PSQ features, particularly in distinguishing between different severity levels of pediatric OSA. The improvements in AUC, sensitivity, and specificity underscore the potential clinical utility of the feature selection approach in enhancing the accuracy of OSA diagnosis.

**Table 3:** Comparison of LDA model performance: PSQ vs. Selected Features (Mean ± SD).

		PSQ features	Our selected features	*P* value
AHI ≥5	**Accuracy**	0.67 ± 0.02	0.68 ± 0.01	0.0763
	**AUC**	0.70 ± 0.02	0.73 ± 0.02	0.0139
	**Sensitivity**	0.48 ± 0.03	0.58 ± 0.03	0.0000
	**Specificity**	0.80 ± 0.02	0.76 ± 0.02	0.0246
	**PPV**	0.62 ± 0.02	0.62 ± 0.03	0.5982
	**NPV**	0.69 ± 0.03	0.72 ± 0.03	0.0007
AHI ≥10	**Balanced Accuracy**	0.57 ± 0.01	0.67 ± 0.01	0.0000
	**AUC**	0.75 ± 0.03	0.78 ± 0.02	0.0238
	**Sensitivity**	0.20 ± 0.04	0.44 ± 0.03	0.0000
	**Specificity**	0.95 ± 0.02	0.90 ± 0.02	0.0008
	**PPV**	0.56 ± 0.12	0.57 ± 0.06	0.6704
	**NPV**	0.80 ± 0.02	0.85 ± 0.01	0.0001

PSQ, Pediatric Sleep Questionnaire; AHI, apnea hypopnea index; AUC, area under the receiver operating characteristic curve; PPV, positive predictive value; NPV, negative predictive value.

The AUC values with the best performance are provided in bold.

## 4 Discussion

The diagnostic ability of six ML methods based on clinical features in children with OSA was assessed in our study. The feature data used in this study can be collected during the day to avoid the burden of physiological signal collection throughout the night on children. The performance of each model was evaluated by the average accuracy rate and AUC value, which were obtained after five repetitions of the data modeling process. The ML models using the dataset with selected features showed slightly better performance compared to those using the dataset with all features. Among the ML models using the selected feature dataset, LDA obtained the best performance with an AUC value of 0.73 and an accuracy rate of 68% for the AHI ≥5 group and an AUC value of 0.78 and an accuracy rate of 67% for the AHI ≥10 group. Classical LDA projects the data onto a lower-dimensional vector space by the projection hyperplane that minimizes the interclass variance and maximizes the distance between the projected means of the classes (38). One possible explanation is the low feature dimension in our study, which gives the LDA model advantages in classification tasks. After feature selection further reduces the number of features, the performance of LDA is further improved.

Sleep questionnaires are the primary method for the daytime diagnosis of OSA in children. Improving its diagnostic performance is a concern of researchers. A recent meta-analysis reported the performance of the PSQ in predicting OSA in children and showed that the accuracy of predicting children with moderate OSA was 62.45%, the sensitivity was 0.79 [95% CI 0.69, 0.86], and the specificity was 0.47 [95% CI 0.28, 0.67]. Therefore, the reliability of the PSQ in real-world clinical populations is still uncertain (50). A recent study used clinical features to identify adult OSA and obtained an AUC value of 0.92, which has an advantage over our predictive model. One possible reason is that part of the predictive characteristics of children come from parents’ description of their children's sleep, which may reduce the reliability of the features (51).

In this study, we observed that specific questions in the original questionnaire were prone to ambiguity, potentially leading to less specific responses from parents. For example, question A3 from the Pediatric Sleep Questionnaire (PSQ), which asks about the frequency of snoring with the descriptor “Always,” might be challenging for parents to interpret accurately. The term “Always” is inherently vague, and parental subjective interpretations may vary, introducing potential inaccuracies in the data. We refined the answers to such questions to address this issue and enhance the precision of responses. Specifically, we focused on the A3 question regarding “Always snore” and introduced a new labeling scheme: Label “0” signifies a negative response, Label “1” corresponds to less than one time per month, Label “2” represents 1–2 times per month, Label “3” indicates 1 to 3 times per week, Label “4” denotes more than three times per week, and Label “5” designates an unclear response. Our research team conducted This refinement process internally, and no direct communication with the questionnaire developers was pursued. While we acknowledge that the original questionnaire designers may have had specific considerations, these adjustments were made within our research team to align with the goals of our study. Through these refinements, we aim to improve the quality and accuracy of the data, ensuring that machine learning algorithms can better comprehend and leverage this information for a more effective classification of pediatric OSA.

Table 4 compares previous studies using machine learning methods to predict OSA in children based on clinical features. Some of these studies have demonstrated that diagnostic models based on machine learning methods and self-built features can offer superior diagnostic accuracy compared to OSA screening questionnaires. For example, Catherine et al. compared the use of self-built features with the PSQ questionnaire in their study and reported the performance of both methods in children with severe OSA. The prediction accuracy of the self-built features was 0.73 (0.64–0.80) compared to that PSQ questionnaire, 0.63 (0.54–0.72) (24). Similarly, Ahmed et al. also used and compared self-built features with five different screening questionnaires, with the self-built model achieving better performance in children with severe OSA (23). The above studies all used data from the Childhood Adenotonsillectomy Trial (CHAT), a randomized controlled trial that compared early outcomes of T&A (52). Our study found similar findings in children with a suspected diagnosis of OSA who went to the outpatient clinic, using an ML algorithm based on 22 features of the PSQ to predict children's level of OSA, and its diagnostic performance was weaker than that of the diagnostic model after feature selection. One possible reason is that the features in the OSA screening questionnaire do not provide enough valid diagnostic information.

**Table 4:** Comparing machine learning methods to predict OSA in children based on clinical features.

Author	Dataset	Age	Features collected	Methodology	Diagnostic cutoff	Accuracy	AUC	Verification
Ahmed et al. (53)	464 Children with OSA from the CHAT	5-10	96	Logistic regression	AHI ≥5 events/h	0.6512	0.6861	Test set
					AHI ≥10 events/h	0.814	0.8721	
Catherine et al. (24)	124 Children with OSA from the CHAT	3-18	22	Linear and logistic regression models	AHI ≥10 events/h (Selected Features)	0.73	-	None
					AHI ≥10 events/h (PSQ-SRDB)	0.65	-	
Our study	2,464 Children with suspected OSA	3-14	102	Logistic regression, Linear discriminant, Radial basis function kernel support vector machine, CatBoost, AdaBoost, Random forest	AHI ≥5 events/h	0.68	0.73	Random test set 5 times
					AHI ≥10 events/h	0.67	0.78	

CHAT, childhood adenotonsillectomy trial; AHI, apnea hypopnea index; AUC, area under the receiver operating characteristic curve; PPV, positive predictive value; NPV, negative predictive value; PSQ-SRBD: pediatric sleep questionnaire-sleep related breathing disorder.

In our study, as shown in Supplementary Material 1, when an AHI of 5 events/h was used as the cutoff value, only 11 of the 22 features of the PSQ questionnaire showed significant differences between individuals with AHI greater than or less than 5 events/h (*P* < 0.05), which meant that half of the features could not benefit diagnosis. In addition, 29 and 34 clinical features from our questionnaire showed significant differences between groups when the AHI cutoff values were 5 and 10 events/h, respectively. Our feature list greatly increased the number of potentially effective features compared with the PSQ questionnaire. We believe it is important to consider the features’ effectiveness when establishing a simple diagnostic tool for OSA based on artificial intelligence. Additionally, due to differences in language and culture, finding effective feature combinations specific to the target population and establishing a diagnostic model may be more suitable than directly using the existing OSA diagnostic questionnaire. Our study is based on non-invasive clinical features. It has a significant cost advantage over traditional sleep monitoring (PSG), which creates the possibility of providing efficient OSA screening tools for children in a resource-limited medical environment. In this context, our model may provide a rapid and economical means for medical teams to identify children with OSA, leading to early intervention and treatment. However, we emphasize that more in-depth research and verification are needed before this method is put into clinical application, and its generalization in different populations, cultures, and medical practices still needs further investigation. We encourage future research teams to conduct more extensive, multicenter research to assess this method's applicability and robustness more comprehensively.

There are some limitations in this study. The AHI cut-off in this study does not include 1 event per hour, which is usually referred to as mild OSA. It should be noted that mild OSA children with persistent symptoms should be recommended for treatment. Although we collected 102 features, we believe not all OSA-related features were collected, for example, other OSA-related features like genetics and environment. Considering other factors affecting OSA, such as genetics and environment, more data types should be added in the future to increase clinical features. In addition, single-center studies, data that may be missing, retrospective design, the age of the participants and the lack of universality of the country may all contribute to data source bias. Although the sample size was larger than that of previous studies, there is still a risk of over-fitting using the same population data for result verification, and future external verification queues are needed to solve this problem. In the machine learning algorithms used in this study, LDA exhibited better performance. One possible reason is that LDA is simple and computationally efficient, which may make it more robust and less prone to overfitting. Nevertheless, we recognize the need for further research to fully elucidate the underlying reasons for the observed performance differences among these classifiers. In addition, more excellent machine learning models such as Bayesian networks should be considered as one of the classification models. In this study, we only focused on traditional feature-based methods for disease classification without exploring the potential of deep learning methods for processing high-dimensional features like images and sounds. With the advantages of deep learning in sleep research (54), introducing deep learning methods into studying OSA disease classification may have potential benefits. Future work can explore deep learning models such as convolution neural networks (CNN) to improve the modeling ability of complex feature relationships.

## 5 Conclusions

In this study, we used ML algorithms to analyze the clinical features of children with a suspected diagnosis of OSA. We found that using ML to investigate clinical features is an effective method to identify OSA in children, and ML models based on clinical features had better predictive ability than ML models based on the PSQ questionnaire. Using the features of nonnocturnal biological signals to stratify the severity of children's OSA is an essential diagnostic supplement to PSG. It provides references for children in areas where PSG is unavailable and cannot be used to determine the severity of OSA. Future research should search for additional practical features that can improve the prediction performance of ML algorithms.

## Data Availability

The raw data supporting the conclusions of this article will be made available by the authors, without undue reservation.
