# Supplementary material for: Pediatric obstructive sleep apnea diagnosis: leveraging machine learning with linear discriminant analysis
Source: Front Pediatr. 2024 Feb 14;12:1328209. doi: 10.3389/fped.2024.1328209 (PMC10899433; doi:10.3389/fped.2024.1328209)
Supplement: Supplementary file 1 [file Table1.docx]

**Supplement Material.1.**

**List of 102 features and its description.**

| **Feature**  **classification** | **Code name** | **Feature** | **Sublabel type** |
| --- | --- | --- | --- |
| Children's personal information and body measurement data | None | Age | E |
|  | None | Sex | H |
|  | None | BMI | E |
|  | None | Height | E |
|  | None | Weight | E |
|  | None | Neck circumference | E |
|  | None | Waist circumference | E |
|  | None | Hip circumference | E |
|  | None | Neck /height ratio | E |
|  | None | Waist/hip ratio | E |
|  | None | Waist /height ratio | E |
|  | None | Hip /height ratio | E |
| Previous history of children | B9 | Did your child stop growing at a normal rate at any time since birth | A |
|  | B22 | Is your child overweight | A |
|  | Q26 | Diagnosed with Allergic rhinitis within the last 1 year | D |
|  | Q27 | Diagnosed with sinusitis within the last 1 year | D |
|  | Q28 | Diagnosed with otitis media within the last 1 year | D |
|  | Q29 | Diagnosed with asthma within the last 1 year | D |
|  | Q30 | Diagnosed with tonsillitis within the last 1 year | D |
|  | Q31 | Diagnosed with sphagitis within the last 1 year | D |
|  | Q39 | Breastfeeding | A |
|  | Q40 | Breastfeeding duration | E |
|  | Q41 | Mixed feeding | A |
|  | Q42 | Duration of breastfeeding when mixed feeding | E |
|  | Q43 | Bottle feeding | A |
|  | None | Born at term | A |
|  | None | Pattern of infant feeding | G |
| Children's daytime performance | A24 | Tend to breathe through the mouth during the day | A |
|  | A25 | Have a dry mouth on waking up in the morning | B |
|  | B7 | Does your child wake up with headaches in the morning | B |
|  | B2 | Have a problem with sleepiness during the day | A |
|  | B4 | Has a teacher commented that your child appears sleepy during the day | A |
|  | C3 | Does not seem to listen when spoken to directly | A |
|  | C5 | Has difficulty organizing tasks and activities | A |
|  | C8 | Is easily distracted by extraneous stimuli | A |
|  | C10 | Fidgets with hands or feet or squirms in seat | A |
|  | C14 | Is “on the go” or often acts as if “driven by a motor” | A |
|  | C18 | Interrupts or intrudes on others | A |
|  | B1 | Wake up feeling unrefreshed in the morning | B |
|  | B6 | It is hard to wake your child up in the morning | A |
|  | Q22 | More active than children of the same age | A |
|  | Q23 | Easy to get angry, difficult to control emotions | A |
|  | Q24 | Academic performance compared to last semester | I |
|  | Q25 | Talk a lot during the day | A |
|  | Q60 | Often doze off while sitting and reading | C |
|  | Q61 | Dozing off frequently while watching TV | C |
|  | Q17 | Often doze off when sitting in public places | C |
|  | Q18 | Dozing off frequently while riding in a car | C |
|  | Q19 | Dozing off frequently in the afternoon | C |
|  | Q20 | Often doze off while sitting and chatting | C |
|  | Q21 | Dozing off frequently after lunch | C |
|  | Q67 | Frequent dozing off when the vehicle is stopped | C |
|  | Q68 | usually study before bed | A |
|  | Q69 | usually watch TV before bed | A |
|  | Q70 | Usually exercising before bed | A |
|  | Q71 | usually make phone calls before bed | A |
|  | Q72 | Usually eat before going to bed | A |
|  | Q73 | Usually read extracurricular books before bed | A |
|  | Q74 | usually play video games before bed | A |
|  | Q75 | Usually exposure to electronic screens before bed | A |
| Children's performance during bedtime | A2 | Snore more than half the time | A |
|  | A3 | Always snore | B |
|  | A4 | Snore loudly | A |
|  | A5 | Have“heavy”or loud breathing | A |
|  | A6 | Have trouble breathing, or struggle to breathe | A |
|  | A7 | Stop breathing during the night | B |
|  | A32 | Occasionally wet the bed | B |
|  | Q1 | time spent in falling asleep | F |
|  | Q2 | time spent to start snoring after falling asleep | F |
|  | Q3 | hard to fall asleep | B |
|  | Q4 | mouth breathing during sleep | B |
|  | Q5 | Cyanosis of lips during sleep | B |
|  | Q6 | sweating while sleeping | B |
|  | Q7 | sleep talking | B |
|  | Q8 | Often tossing and turning in sleep | B |
|  | Q9 | somnambulate | B |
|  | Q10 | grind teeth in sleep | B |
|  | Q11 | night terror | B |
|  | Q12 | Mouth foam during sleep | B |
|  | Q13 | feeling sleepy after waking up | B |
|  | Q14 | shaking head when about to fall asleep | B |
| Characteristics of family members | Q32 | Whether family members snore | A |
|  | Q33 | Does the father snore | A |
|  | Q34 | Does the mother snore | A |
|  | Q35 | Does the grandfather snore | A |
|  | Q36 | Does the grandmother snore | A |
|  | Q37 | Does the maternal grandmother snore | A |
|  | Q38 | Does the maternal grandfather snore | A |
|  | Q44 | The educational Level of father | J |
|  | Q45 | The educational Level of mother | J |
|  | Q46 | Whether family members smoke | A |
|  | Q47 | Does the father smoke | A |
|  | Q48 | Does the mother smoke | A |
|  | Q49 | Does the grandfather smoke | A |
|  | Q50 | Does the grandmother smoke | A |
|  | Q51 | Does the maternal grandmother smoke | A |
|  | Q52 | Does the maternal grandfather smoke | A |
|  | Q53 | Whether family members diagnosed with sleep apnea | A |
|  | Q54 | Has the father been diagnosed with sleep apnea | A |
|  | Q55 | Has the mother been diagnosed with sleep apnea | A |
|  | Q56 | Has the grandfather been diagnosed with sleep apnea | A |
|  | Q57 | Has the grandmother been diagnosed with sleep apnea | A |
|  | Q58 | Has the maternal grandmother been diagnosed with sleep apnea | A |
|  | Q59 | Has the maternal grandfather been diagnosed with sleep apnea | A |
| Meaning of sublabel type | | | |
| A | Label"0" means negative answer, label "1" means affirmative answer. | | |
| B | Label"0" means negative answer, label"1" means less than 1 time per month, label"2" means 1 to 2 times per month, label"3" means 1 to 3 times per week, label"4" means more than 3 times per week, label"5" means unclear. | | |
| C | Label"0" means negative answer, label"1" means less than 1 time per month, label"2" means 1 to 2 times per month, label"3" means 1 to 2 times per week, and label"4" means 1 to 2 times per day,label"5" means more than 2 times a day. | | |
| D | Label "0" means negative answer, label"1" means 1 to 3 times per year, and label"2" means 4 times or more per year. | | |
| E | Label numbers are actual values. | | |
| F | Label "1" means less than 10 minutes, label "2" means 10-30 minutes, label "3" means 31-60 minutes, and label "4" means more than 60 minutes. | | |
| G | Label "1" means exclusive breastfeeding, label "2" means mixed feeding, and label "3" means artificial feeding. | | |
| H | Label "1" means male, label "2" means female. | | |
| I | Label "1" means progress, label "2" means equal, label "3" means backward. | | |
| J | Label "1" means none, label "2" means elementary school, label "3" means junior high school, label "4" means high school, label "5" means undergraduate, label "6" means master's degree or above. | | |

| **Comparison of clinical features of negative OSA and positive OSA in children.**  Group comparison was conducted using the Chi-squared test or the Student’s t-test*. P-value<0.05 was considered significant. Data are expressed as Mean(SD). The feature in Pediatric Sleep Questionnaire (PSQ) is A2, A3, A4, A5, A6, A7, A24, A25, B7, B2, B4, C3, C5, C8, C10 ,C14, C18, A32, B1, B6, B9, B22. | | | | | | |
| --- | --- | --- | --- | --- | --- | --- |
|  | **AHI≥5 as the cut-off value** | | | **AHI≥10 as the cut-off value** | | |
| Code name | Negative | Positive | P-values | Negative | Positive | P-values |
| A2 | 0.25(0.43) | 0.46(0.5) | 0.000 | 0.28(0.45) | 0.55(0.5) | 0.000 |
| A3 | 3.01(1.2) | 3.54(0.88) | 0.000 | 3.11(1.15) | 3.63(0.84) | 0.000 |
| A4 | 0.39(0.49) | 0.6(0.49) | 0.000 | 0.42(0.49) | 0.68(0.47) | 0.000 |
| A5 | 0.61(0.49) | 0.68(0.47) | 0.000 | 0.61(0.49) | 0.71(0.45) | 0.000 |
| A6 | 0.32(0.47) | 0.51(0.5) | 0.000 | 0.35(0.48) | 0.58(0.49) | 0.000 |
| A7 | 0.77(1.26) | 1.37(1.56) | 0.000 | 0.83(1.3) | 1.67(1.65) | 0.000 |
| A24 | 0.27(0.44) | 0.24(0.43) | 0.102 | 0.26(0.44) | 0.24(0.43) | 0.228 |
| A25 | 0.92(1.41) | 0.95(1.42) | 0.628 | 0.9(1.39) | 1.03(1.48) | 0.220 |
| B7 | 0.13(0.53) | 0.13(0.53) | 0.931 | 0.13(0.53) | 0.13(0.53) | 0.988 |
| B2 | 0.04(0.18) | 0.05(0.21) | 0.147 | 0.03(0.18) | 0.06(0.23) | 0.016 |
| B4 | 0.04(0.19) | 0.05(0.21) | 0.428 | 0.04(0.19) | 0.06(0.23) | 0.035 |
| C3 | 0.36(0.48) | 0.29(0.45) | 0.000 | 0.34(0.48) | 0.27(0.45) | 0.002 |
| C5 | 0.17(0.38) | 0.17(0.38) | 0.949 | 0.17(0.38) | 0.16(0.36) | 0.382 |
| C8 | 0.51(0.5) | 0.49(0.5) | 0.278 | 0.5(0.5) | 0.49(0.5) | 0.516 |
| C10 | 0.3(0.46) | 0.26(0.44) | 0.072 | 0.29(0.46) | 0.25(0.44) | 0.068 |
| C14 | 0.32(0.47) | 0.28(0.45) | 0.037 | 0.31(0.47) | 0.26(0.44) | 0.018 |
| C18 | 0.39(0.49) | 0.33(0.47) | 0.002 | 0.37(0.48) | 0.33(0.47) | 0.067 |
| A32 | 0.36(0.88) | 0.45(1) | 0.023 | 0.38(0.9) | 0.48(1.06) | 0.007 |
| B1 | 0.56(1.08) | 0.55(1.11) | 0.342 | 0.55(1.07) | 0.58(1.16) | 0.289 |
| B6 | 0.15(0.35) | 0.15(0.36) | 0.614 | 0.15(0.35) | 0.16(0.37) | 0.418 |
| B9 | 0.15(0.35) | 0.13(0.34) | 0.240 | 0.15(0.36) | 0.1(0.3) | 0.004 |
| B22 | 0.23(0.42) | 0.4(0.49) | 0.000 | 0.25(0.43) | 0.48(0.5) | 0.000 |
| Q1 | 2.08(0.65) | 2(0.61) | 0.017 | 2.07(0.64) | 1.96(0.6) | 0.004 |
| Q2 | 2.61(1.17) | 2.25(1.12) | 0.000 | 2.56(1.17) | 2.1(1.08) | 0.000 |
| Q3 | 0.75(1.19) | 0.61(1.12) | 0.039 | 0.73(1.19) | 0.56(1.07) | 0.057 |
| Q4 | 3.01(1.39) | 3.39(1.16) | 0.000 | 3.07(1.36) | 3.5(1.08) | 0.000 |
| Q5 | 0.07(0.39) | 0.1(0.51) | 0.234 | 0.07(0.41) | 0.12(0.56) | 0.132 |
| Q6 | 2.13(1.7) | 2.15(1.72) | 0.868 | 2.14(1.7) | 2.13(1.73) | 0.776 |
| Q7 | 0.82(1.05) | 0.75(1.05) | 0.118 | 0.82(1.06) | 0.71(1.03) | 0.089 |
| Q8 | 2.47(1.58) | 2.64(1.59) | 0.001 | 2.5(1.59) | 2.66(1.6) | 0.043 |
| Q9 | 0.05(0.32) | 0.04(0.25) | 0.245 | 0.05(0.31) | 0.03(0.21) | 0.439 |
| Q10 | 0.77(1.25) | 0.63(1.14) | 0.041 | 0.75(1.23) | 0.59(1.11) | 0.045 |
| Q11 | 0.44(0.83) | 0.36(0.73) | 0.134 | 0.41(0.79) | 0.39(0.8) | 0.824 |
| Q12 | 0.16(0.62) | 0.36(0.97) | 0.000 | 0.19(0.71) | 0.41(1.02) | 0.000 |
| Q13 | 0.72(1.23) | 0.72(1.23) | 0.949 | 0.72(1.22) | 0.72(1.25) | 0.906 |
| Q14 | 0.12(0.53) | 0.11(0.48) | 0.421 | 0.13(0.53) | 0.09(0.44) | 0.021 |
| Q15 | 0.23(0.71) | 0.23(0.72) | 0.022 | 0.22(0.7) | 0.25(0.77) | 0.324 |
| Q16 | 0.13(0.48) | 0.12(0.45) | 0.298 | 0.13(0.48) | 0.11(0.45) | 0.810 |
| Q17 | 0.11(0.46) | 0.11(0.43) | 0.882 | 0.11(0.45) | 0.11(0.43) | 0.938 |
| Q18 | 0.96(1.3) | 0.84(1.2) | 0.225 | 0.94(1.28) | 0.83(1.17) | 0.367 |
| Q19 | 0.45(0.99) | 0.39(0.94) | 0.367 | 0.44(0.97) | 0.39(0.97) | 0.313 |
| Q20 | 0.07(0.35) | 0.06(0.31) | 0.735 | 0.07(0.35) | 0.05(0.27) | 0.382 |
| Q21 | 0.42(1.01) | 0.37(0.91) | 0.222 | 0.41(0.98) | 0.37(0.94) | 0.099 |
| Q22 | 0.29(0.45) | 0.26(0.44) | 0.142 | 0.29(0.45) | 0.26(0.44) | 0.174 |
| Q23 | 0.29(0.46) | 0.25(0.44) | 0.035 | 0.28(0.45) | 0.25(0.43) | 0.109 |
| Q24 | 2.49(0.85) | 2.45(0.87) | 0.492 | 2.48(0.86) | 2.47(0.85) | 0.592 |
| Q25 | 0.41(0.49) | 0.38(0.49) | 0.257 | 0.41(0.49) | 0.37(0.48) | 0.104 |
| Q26 | 0.86(0.84) | 0.79(0.83) | 0.129 | 0.85(0.84) | 0.75(0.82) | 0.045 |
| Q27 | 0.33(0.62) | 0.28(0.61) | 0.007 | 0.32(0.63) | 0.26(0.59) | 0.022 |
| Q28 | 0.16(0.41) | 0.14(0.37) | 0.272 | 0.16(0.41) | 0.13(0.37) | 0.446 |
| Q29 | 0.06(0.28) | 0.05(0.25) | 0.423 | 0.06(0.27) | 0.06(0.25) | 0.372 |
| Q30 | 0.62(0.73) | 0.65(0.73) | 0.359 | 0.61(0.73) | 0.71(0.76) | 0.034 |
| Q31 | 0.53(0.68) | 0.54(0.7) | 0.473 | 0.53(0.69) | 0.56(0.71) | 0.426 |
| Q32 | 0.78(0.41) | 0.76(0.43) | 0.212 | 0.77(0.42) | 0.77(0.42) | 0.970 |
| Q33 | 0.55(0.5) | 0.56(0.5) | 0.793 | 0.55(0.5) | 0.55(0.5) | 0.910 |
| Q34 | 0.12(0.33) | 0.11(0.31) | 0.332 | 0.12(0.32) | 0.11(0.32) | 0.752 |
| Q35 | 0.19(0.39) | 0.17(0.38) | 0.335 | 0.18(0.39) | 0.18(0.39) | 0.989 |
| Q36 | 0.14(0.35) | 0.11(0.32) | 0.037 | 0.13(0.34) | 0.12(0.33) | 0.451 |
| Q37 | 0.11(0.31) | 0.09(0.28) | 0.099 | 0.11(0.31) | 0.07(0.25) | 0.010 |
| Q38 | 0.14(0.35) | 0.12(0.33) | 0.119 | 0.14(0.35) | 0.12(0.33) | 0.327 |
| Q39 | 0.54(0.5) | 0.55(0.5) | 0.446 | 0.53(0.5) | 0.57(0.5) | 0.176 |
| Q40 | 1.87(1.94) | 1.9(1.91) | 0.620 | 1.86(1.92) | 1.98(1.93) | 0.794 |
| Q41 | 0.32(0.47) | 0.31(0.46) | 0.788 | 0.32(0.47) | 0.29(0.45) | 0.101 |
| Q42 | 0.81(1.34) | 0.84(1.41) | 0.516 | 0.84(1.37) | 0.76(1.34) | 0.553 |
| Q43 | 0.15(0.35) | 0.13(0.34) | 0.399 | 0.14(0.35) | 0.14(0.35) | 0.904 |
| Q44 | 4.9(0.84) | 4.74(0.93) | 0.002 | 4.88(0.84) | 4.65(0.98) | 0.000 |
| Q45 | 4.87(0.82) | 4.7(0.9) | 0.000 | 4.86(0.82) | 4.6(0.95) | 0.000 |
| Q46 | 0.42(0.49) | 0.45(0.5) | 0.088 | 0.42(0.49) | 0.46(0.5) | 0.067 |
| Q47 | 0.32(0.47) | 0.34(0.47) | 0.470 | 0.33(0.47) | 0.33(0.47) | 0.703 |
| Q48 | 0.08(0.28) | 0.08(0.28) | 0.920 | 0.08(0.28) | 0.09(0.29) | 0.601 |
| Q49 | 0.09(0.29) | 0.1(0.3) | 0.542 | 0.09(0.29) | 0.11(0.31) | 0.227 |
| Q50 | 0.04(0.2) | 0.05(0.23) | 0.103 | 0.04(0.2) | 0.06(0.24) | 0.032 |
| Q51 | 0.03(0.16) | 0.03(0.18) | 0.349 | 0.03(0.17) | 0.03(0.16) | 0.765 |
| Q52 | 0.06(0.24) | 0.05(0.23) | 0.495 | 0.06(0.24) | 0.06(0.23) | 0.946 |
| Q53 | 0.06(0.25) | 0.07(0.26) | 0.338 | 0.07(0.25) | 0.07(0.26) | 0.534 |
| Q54 | 0.04(0.19) | 0.05(0.21) | 0.206 | 0.04(0.19) | 0.05(0.22) | 0.293 |
| Q55 | 0(0.06) | 0(0.06) | 0.953 | 0(0.06) | 0.01(0.08) | 0.526 |
| Q56 | 0.01(0.08) | 0.01(0.09) | 0.431 | 0.01(0.07) | 0.01(0.12) | 0.019 |
| Q57 | 0(0.06) | 0(0.06) | 0.829 | 0(0.06) | 0(0.04) | 0.438 |
| Q58 | 0.01(0.08) | 0(0.06) | 0.456 | 0.01(0.08) | 0(0.04) | 0.218 |
| Q59 | 0.01(0.12) | 0.01(0.1) | 0.449 | 0.01(0.12) | 0.01(0.09) | 0.202 |
| Q60 | 0.23(0.71) | 0.23(0.72) | 0.022 | 0.22(0.7) | 0.25(0.77) | 0.324 |
| Q61 | 0.13(0.48) | 0.12(0.45) | 0.298 | 0.13(0.48) | 0.11(0.45) | 0.810 |
| Q62 | 0.11(0.46) | 0.11(0.43) | 0.882 | 0.11(0.45) | 0.11(0.43) | 0.938 |
| Q63 | 0.96(1.3) | 0.84(1.2) | 0.225 | 0.94(1.28) | 0.83(1.17) | 0.367 |
| Q64 | 0.45(0.99) | 0.39(0.94) | 0.367 | 0.44(0.97) | 0.39(0.97) | 0.313 |
| Q65 | 0.07(0.35) | 0.06(0.31) | 0.735 | 0.07(0.35) | 0.05(0.27) | 0.382 |
| Q66 | 0.42(1.01) | 0.37(0.91) | 0.222 | 0.41(0.98) | 0.37(0.94) | 0.099 |
| Q67 | 0.34(0.85) | 0.33(0.87) | 0.662 | 0.35(0.88) | 0.27(0.77) | 0.163 |
| Q68 | 0.33(0.47) | 0.34(0.47) | 0.761 | 0.33(0.47) | 0.36(0.48) | 0.195 |
| Q69 | 0.52(0.5) | 0.57(0.5) | 0.011 | 0.53(0.5) | 0.59(0.49) | 0.006 |
| Q70 | 0.12(0.33) | 0.13(0.33) | 0.844 | 0.13(0.33) | 0.12(0.33) | 0.974 |
| Q71 | 0(0.07) | 0.01(0.09) | 0.326 | 0.01(0.08) | 0.01(0.08) | 0.869 |
| Q72 | 0.18(0.38) | 0.14(0.35) | 0.011 | 0.17(0.38) | 0.13(0.34) | 0.039 |
| Q73 | 0.59(0.49) | 0.55(0.5) | 0.075 | 0.59(0.49) | 0.52(0.5) | 0.006 |
| Q74 | 0.09(0.29) | 0.11(0.31) | 0.135 | 0.09(0.29) | 0.12(0.33) | 0.035 |
| Q75 | 0.02(0.13) | 0.02(0.13) | 0.694 | 0.02(0.13) | 0.01(0.12) | 0.726 |
| age | 5.76(2.4) | 5.92(2.39) | 0.165 | 5.73(2.35) | 6.15(2.53) | 0.015 |
| sex | 1.36(0.48) | 1.27(0.45) | 0.000 | 1.35(0.48) | 1.24(0.43) | 0.000 |
| BMI | 16.07(3.31) | 17.69(4.63) | 0.000* | 16.21(3.45) | 18.61(5.07) | 0.000* |
| Born_at_term | 0.95(0.21) | 0.93(0.25) | 0.024 | 0.94(0.23) | 0.94(0.23) | 0.834 |
| pattern_of_infant_feeding | 1.61(0.73) | 1.58(0.72) | 0.630 | 1.61(0.72) | 1.57(0.73) | 0.260 |
| height | 120.38(16.38) | 122.78(16.89) | 0.000* | 120.41(16.21) | 124.79(17.67) | 0.000* |
| weight | 24.53(12) | 28.49(15.38) | 0.000* | 24.76(12.1) | 31.15(17.17) | 0.000* |
| Neck_circumference | 26.91(2.89) | 28.04(3.68) | 0.000* | 26.98(2.91) | 28.81(4.05) | 0.000* |
| Waist_circumference | 58.57(9.18) | 62.7(12.55) | 0.000* | 58.88(9.44) | 65.21(13.87) | 0.000* |
| Hip_circumference | 65.29(10.75) | 68.88(13.19) | 0.000* | 65.52(10.92) | 71.22(14.18) | 0.000* |
| Neck/height_ratio | 0.225(0.02) | 0.229(0.02) | 0.000* | 0.226(0.02) | 0.232(0.02) | 0.000* |
| Waist/hip_ratio | 0.9(0.05) | 0.911(0.05) | 0.000* | 0.901(0.05) | 0.915(0.05) | 0.000* |
| Waist/height_ratio | 0.488(0.05) | 0.51(0.06) | 0.000* | 0.49(0.05) | 0.521(0.07) | 0.000* |
| Hip/height_ratio | 0.542(0.04) | 0.559(0.05) | 0.000* | 0.544(0.04) | 0.568(0.06) | 0.000* |
