# Supplementary material for: Pediatric obstructive sleep apnea diagnosis: leveraging machine learning with linear discriminant analysis
Source: Front Pediatr. 2024 Feb 14;12:1328209. doi: 10.3389/fped.2024.1328209 (PMC10899433; doi:10.3389/fped.2024.1328209)
Supplement: Supplementary file 2 [file Table2.docx]

**Supplement Material.2.** **The feature selection procedure and the list of the optimal alpha and L1_ratio values used for the elastic net.**

In each iteration, we employed a different random seed value for data splitting during raw data preprocessing. The elastic net was fixed on the preprocessed datasets, and an optimal alpha and L1_ratio value were calculated through 10 repetitions of 5-fold cross-validation. This process was repeated five times using five different random seeds.

Using the five optimal alpha and L1_ratio values, we computed the feature coefficients with the elastic net, resulting in five feature sets of varying sizes. These feature sets included features with coefficients greater than zero. Finally, we obtained a new and reliable feature set by identifying the common features among the five sets.

In this study, the 5-kfold cross-validation involved dividing the training set into five equal-sized random partitions. Four partitions were used for training the model, and one partition was used for validation.

| **the binary classification threshold** | **parameter type of the elastic net** | **the serial number of 5 repeation when using 5 different random seed value for data splitting** | | | | |
| --- | --- | --- | --- | --- | --- | --- |
|  |  | **1** | **2** | **3** | **4** | **5** |
|  |  | random seed=786 | random seed=256 | random seed=321 | random seed=423 | random seed=521 |
| an AHI of 5 events/h | the optimal alpha | 0.01 | 0.01 | 0.01 | 0.01 | 0.01 |
|  | the optimal L1_ratio | 0.37 | 0.36 | 0.38 | 0.37 | 0.36 |
| an AHI of 10 events/h | the optimal alpha | 0.1 | 0.1 | 0.1 | 0.1 | 0.1 |
|  | the optimal L1_ratio | 0.01 | 0.01 | 0.01 | 0.01 | 0.01 |
