# Supplementary material for: Pediatric obstructive sleep apnea diagnosis: leveraging machine learning with linear discriminant analysis
Source: Front Pediatr. 2024 Feb 14;12:1328209. doi: 10.3389/fped.2024.1328209 (PMC10899433; doi:10.3389/fped.2024.1328209)
Supplement: Supplementary file 3 [file Table3.docx]

| **Supplement Material.3. The list of the selected features** | | | | | | | | |
| --- | --- | --- | --- | --- | --- | --- | --- | --- |
| **Cut-off value** | **No.** | **Features Name** | **No.** | **Features Name** | **No.** | **Features Name** | **No.** | **Features Name** |
| **AHI≥5** | **1** | A2 | **9** | Q3 | **17** | Q27 | **25** | C10 |
|  | **2** | A3 | **10** | Q4 | **18** | Q30 | **26** | C14 |
|  | **3** | A4 | **11** | Q6 | **19** | Q45 | **27** | Neck circumference |
|  | **4** | A6 | **12** | Q7 | **20** | Q69 | **28** | Hip circumference |
|  | **5** | A7 | **13** | Q10 | **21** | Q72 | **29** | Neck/height_ratio |
|  | **6** | A24 | **14** | Q19 | **22** | B22 | **30** | Waist/hip_ratio |
|  | **7** | A25 | **15** | Q23 | **23** | C3 | **31** | sex |
|  | **8** | Q2 | **16** | Q26 | **24** | C5 |  |  |
| **AHI≥10** | **1** | A2 | **13** | Q6 | **25** | Q27 | **37** | C3 |
|  | **2** | A3 | **14** | Q7 | **26** | Q30 | **38** | C10 |
|  | **3** | A4 | **15** | Q8 | **27** | Q31 | **39** | C18 |
|  | **4** | A6 | **16** | Q10 | **28** | Q40 | **40** | Neck circumference |
|  | **5** | A7 | **17** | Q11 | **29** | Q42 | **41** | Hip circumference |
|  | **6** | A24 | **18** | Q13 | **30** | Q44 | **42** | Neck/height_ratio |
|  | **7** | A25 | **19** | Q18 | **31** | Q45 | **43** | Waist/hip_ratio |
|  | **8** | A32 | **20** | Q19 | **32** | Q60 | **44** | Waist/height_ratio |
|  | **9** | Q1 | **21** | Q21 | **33** | Q67 | **45** | Born_at_term |
|  | **10** | Q2 | **22** | Q24 | **34** | Q72 | **46** | sex |
|  | **11** | Q3 | **23** | Q25 | **35** | B1 | **47** | age |
|  | **12** | Q4 | **24** | Q26 | **36** | B22 |  |  |
