# Supplementary material for: Pediatric obstructive sleep apnea diagnosis: leveraging machine learning with linear discriminant analysis
Source: Front Pediatr. 2024 Feb 14;12:1328209. doi: 10.3389/fped.2024.1328209 (PMC10899433; doi:10.3389/fped.2024.1328209)
Supplement: Supplementary file 4 [file Table4.docx]

**Supplement Material.4. The absolute value of the feature coefficient obtained by elastic net with the optimal alpha and L1_ratio value.**

| AHI≥5 as the cut-off value | | | | | | | | | |
| --- | --- | --- | --- | --- | --- | --- | --- | --- | --- |
| Random Seed=786 | | Random Seed=256 | | Random Seed=321 | | Random Seed=423 | | Random Seed=521 | |
| Variable | Value | Variable | Value | Variable | Value | Variable | Value | Variable | Value |
| A3_4 | 0.081 | A3_4 | 0.082 | A3_4 | 0.082 | A3_4 | 0.082 | A3_4 | 0.084 |
| A6_1 | 0.074 | A6_1 | 0.075 | A6_1 | 0.075 | A6_1 | 0.075 | A6_1 | 0.075 |
| A2_1 | 0.063 | A7_0 | 0.063 | A7_0 | 0.063 | A7_0 | 0.063 | A7_0 | 0.063 |
| A7_0 | 0.062 | A2_0 | 0.059 | A2_0 | 0.059 | A2_0 | 0.059 | A2_0 | 0.06 |
| A4_0 | 0.052 | A4_1 | 0.057 | A4_1 | 0.057 | A4_1 | 0.057 | A4_0 | 0.055 |
| Q12_0 | 0.052 | Q4_4 | 0.05 | Q4_4 | 0.05 | Q4_4 | 0.05 | Q4_4 | 0.05 |
| Q4_4 | 0.049 | A7_4 | 0.045 | A7_4 | 0.046 | A7_4 | 0.046 | A7_4 | 0.046 |
| A7_4 | 0.045 | B22_0 | 0.04 | B22_1 | 0.04 | B22_1 | 0.04 | B22_0 | 0.04 |
| B22_1 | 0.039 | sex_2 | 0.037 | sex_1 | 0.037 | sex_2 | 0.037 | sex_1 | 0.037 |
| sex_1 | 0.038 | A3_2 | 0.034 | A24_1 | 0.035 | A24_1 | 0.035 | A3_2 | 0.033 |
| A24_1 | 0.036 | A24_0 | 0.033 | A3_2 | 0.034 | A3_2 | 0.034 | A24_0 | 0.033 |
| A3_2 | 0.035 | C3_0 | 0.032 | C3_1 | 0.03 | C3_0 | 0.032 | C3_0 | 0.032 |
| C3_0 | 0.031 | Q3_0 | 0.028 | Q3_0 | 0.028 | Q3_0 | 0.028 | Q3_0 | 0.028 |
| Q3_0 | 0.029 | Q4_1 | 0.027 | Q4_1 | 0.028 | Q4_1 | 0.027 | Q4_1 | 0.028 |
| Q11_0 | 0.028 | Q11_0 | 0.027 | Q11_0 | 0.026 | Q11_0 | 0.027 | Q11_0 | 0.027 |
| Q4_1 | 0.027 | A32_0 | 0.026 | A32_0 | 0.026 | A32_0 | 0.026 | A32_0 | 0.026 |
| Q12_4 | 0.026 | Waist/height_ratio | 0.026 | Waist/height_ratio | 0.025 | Waist/height_ratio | 0.026 | Q19_0 | 0.025 |
| Q60_3 | 0.026 | Q19_0 | 0.025 | Q60_3 | 0.025 | Q19_0 | 0.025 | Waist/height_ratio | 0.025 |
| Q10_4 | 0.025 | Q72_1 | 0.025 | Q19_0 | 0.025 | Q72_0 | 0.024 | Q72_1 | 0.025 |
| A32_0 | 0.025 | Q10_4 | 0.024 | Q72_1 | 0.024 | Q60_3 | 0.024 | Q27_1 | 0.025 |
| Q27_1 | 0.025 | Q27_0 | 0.024 | Q10_4 | 0.024 | Q10_4 | 0.024 | Q27_0 | 0.025 |
| Q27_0 | 0.025 | Q27_1 | 0.024 | Q27_0 | 0.024 | Q27_0 | 0.024 | Q10_4 | 0.024 |
| Q19_0 | 0.024 | Q60_3 | 0.024 | Q27_1 | 0.024 | Q27_1 | 0.024 | Q60_3 | 0.024 |
| Waist/height_ratio | 0.024 | Born_at_term_1 | 0.024 | Born_at_term_1 | 0.024 | Born_at_term_1 | 0.024 | Q4_3 | 0.024 |
| Q72_1 | 0.024 | Q4_3 | 0.024 | C18_0 | 0.024 | Q4_3 | 0.023 | Born_at_term_1 | 0.024 |
| Q4_3 | 0.024 | C18_0 | 0.023 | Q4_3 | 0.024 | C18_1 | 0.023 | C18_0 | 0.023 |
| Born_at_term_1 | 0.023 | Waist/hip_ratio | 0.022 | Waist/hip_ratio | 0.023 | C10_1 | 0.022 | Waist/hip_ratio | 0.022 |
| C18_1 | 0.023 | C10_0 | 0.021 | C10_1 | 0.022 | Waist/hip_ratio | 0.022 | C10_0 | 0.021 |
| Waist/hip_ratio | 0.023 | A3_0 | 0.021 | A3_0 | 0.021 | A3_0 | 0.021 | Q8_4 | 0.02 |
| C10_1 | 0.021 | Q8_4 | 0.02 | Q8_4 | 0.02 | Q8_4 | 0.02 | Q4_0 | 0.02 |
| Q8_4 | 0.02 | Q4_0 | 0.02 | C14_0 | 0.02 | Q4_0 | 0.019 | C14_1 | 0.019 |
| Q2_4 | 0.019 | Q2_4 | 0.019 | Q4_0 | 0.019 | Q2_4 | 0.019 | age_7 | 0.018 |
| Hip_circumference | 0.019 | C14_1 | 0.019 | Q2_4 | 0.019 | C14_1 | 0.018 | Q30_1 | 0.018 |
| Q4_0 | 0.019 | age_7 | 0.018 | age_7 | 0.018 | age_7 | 0.018 | Q24_1 | 0.018 |
| age_7 | 0.019 | Q30_1 | 0.018 | Q30_1 | 0.018 | Q30_1 | 0.018 | Q4_2 | 0.017 |
| C14_1 | 0.018 | Q24_1 | 0.018 | Q24_1 | 0.018 | Q24_1 | 0.018 | Hip_circumference | 0.017 |
| Neck_circumference | 0.017 | Q4_2 | 0.018 | Hip_circumference | 0.018 | Q4_2 | 0.018 | Q2_4 | 0.017 |
| Q30_1 | 0.017 | Hip_circumference | 0.017 | Q4_2 | 0.018 | Neck_circumference | 0.017 | Neck_circumference | 0.017 |
| A3_0 | 0.017 | Neck_circumference | 0.017 | Neck_circumference | 0.017 | Hip_circumference | 0.017 | Q45_6 | 0.017 |
| Q45_6 | 0.017 | Q45_6 | 0.017 | Q45_6 | 0.017 | Q45_6 | 0.017 | age_6 | 0.016 |
| Q4_2 | 0.017 | Q36_0 | 0.016 | Q36_1 | 0.016 | Q36_0 | 0.016 | Q24_3 | 0.016 |
| Q24_1 | 0.017 | age_6 | 0.016 | age_6 | 0.016 | age_6 | 0.016 | Q36_0 | 0.016 |
| age_6 | 0.016 | Q24_3 | 0.016 | Q24_3 | 0.016 | Q24_3 | 0.015 | Q7_0 | 0.015 |
| Q36_0 | 0.015 | Q7_0 | 0.015 | Q7_0 | 0.015 | Q7_0 | 0.015 | Q23_0 | 0.014 |
| Q24_3 | 0.015 | Q11_2 | 0.014 | Q23_0 | 0.014 | Q23_0 | 0.014 | Q11_2 | 0.014 |
| Q7_0 | 0.015 | Q45_3 | 0.014 | Q45_3 | 0.014 | Q45_3 | 0.014 | Q2_2 | 0.014 |
| Q2_ | 0.014 | Q70_0 | 0.014 | Q11_2 | 0.014 | Q11_2 | 0.014 | Q45_3 | 0.014 |
| Q11_2 | 0.014 | Q23_1 | 0.014 | Q32_1 | 0.014 | Q70_0 | 0.014 | Q70_1 | 0.014 |
| Q19_3 | 0.013 | Q43_1 | 0.013 | Q70_0 | 0.013 | Q32_1 | 0.014 | Q32_1 | 0.013 |
| A25_4 | 0.013 | Q19_3 | 0.013 | Q43_0 | 0.013 | Q43_0 | 0.013 | Q10_0 | 0.013 |
| Q70_1 | 0.013 | Q18_1 | 0.013 | age_5 | 0.013 | age_5 | 0.013 | age_5 | 0.013 |
| Q45_3 | 0.013 | A25_3 | 0.013 | Q10_0 | 0.013 | Q10_0 | 0.013 | Neck/height_ratio | 0.013 |
| Q10_0 | 0.013 | age_5 | 0.013 | A25_3 | 0.012 | Q19_3 | 0.013 | Q19_3 | 0.013 |
| pattern_of_infant_feeding_3 | 0.013 | Q10_0 | 0.013 | Q18_1 | 0.012 | Q18_1 | 0.012 | Q18_1 | 0.012 |
| Q18_1 | 0.012 | Neck/height_ratio | 0.012 | Q19_3 | 0.012 | A25_3 | 0.012 | Q2_3 | 0.012 |
| age_5 | 0.012 | Q2_2 | 0.012 | Neck/height_ratio | 0.012 | Q46_1 | 0.012 | A25_3 | 0.012 |
| Neck/height_ratio | 0.012 | A7_1 | 0.012 | A7_1 | 0.012 | Neck/height_ratio | 0.012 | A7_1 | 0.012 |
| age_3 | 0.012 | Q46_0 | 0.012 | Q46_0 | 0.012 | A7_1 | 0.012 | Q46_0 | 0.012 |
| A7_1 | 0.012 | age_3 | 0.011 | Q2_2 | 0.012 | Q2_2 | 0.012 | age_3 | 0.012 |
| Q32_0 | 0.012 | Q26_2 | 0.011 | Q26_2 | 0.012 | Q69_0 | 0.011 | Q40_4 | 0.012 |
| Q46_1 | 0.012 | Q44_3 | 0.011 | age_3 | 0.011 | Q26_2 | 0.011 | Q69_1 | 0.011 |
| Q26_2 | 0.012 | Q40_4 | 0.011 | Q44_3 | 0.011 | age_3 | 0.011 | Q26_2 | 0.011 |
| A25_3 | 0.012 | Q69_1 | 0.011 | Q69_1 | 0.011 | Q44_3 | 0.011 | Q44_3 | 0.011 |
| A3_1 | 0.011 | Q11_1 | 0.011 | Q40_4 | 0.011 | Q40_4 | 0.011 | Q21_1 | 0.011 |
| Q21_1 | 0.011 | Q32_0 | 0.011 | Q21_1 | 0.011 | A3_1 | 0.011 | Q40_2 | 0.011 |
| Q44_3 | 0.011 | A3_1 | 0.011 | A32_2 | 0.011 | Q21_1 | 0.011 | Q11_1 | 0.011 |
| A32_2 | 0.011 | Q13_0 | 0.011 | A3_1 | 0.011 | A32_2 | 0.011 | A25_4 | 0.011 |
| Q40_4 | 0.011 | A32_2 | 0.011 | A25_4 | 0.011 | A25_4 | 0.011 | Q8_2 | 0.011 |
| Q69_0 | 0.011 | Q2_3 | 0.011 | Q2_3 | 0.011 | Q11_1 | 0.011 | A32_2 | 0.011 |
| Q40_2 | 0.01 | Q7_2 | 0.01 | Q11_1 | 0.011 | Q2_3 | 0.011 | Q13_0 | 0.011 |
| Q2_3 | 0.01 | A25_2 | 0.01 | Q13_0 | 0.01 | Q13_0 | 0.01 | Q7_2 | 0.011 |
| Q7_2 | 0.01 | Q44_4 | 0.01 | Q8_2 | 0.01 | Q22_1 | 0.01 | Q44_4 | 0.01 |
| Q44_4 | 0.01 | A25_4 | 0.01 | Q7_2 | 0.01 | Q7_2 | 0.01 | Q60_1 | 0.01 |
| Q11_1 | 0.01 | Q8_2 | 0.01 | Q44_4 | 0.01 | Q44_4 | 0.01 | A3_1 | 0.01 |
| Q8_2 | 0.01 | Q40_2 | 0.01 | Q40_2 | 0.01 | Q8_2 | 0.01 | Q22_0 | 0.01 |
| Q60_1 | 0.01 | Q22_0 | 0.01 | Q22_0 | 0.01 | Q60_1 | 0.01 | Q18_3 | 0.01 |
| Q6_4 | 0.01 | Q60_1 | 0.01 | Q18_3 | 0.01 | Q40_2 | 0.01 | Q17_0 | 0.01 |
| A25_2 | 0.01 | Q18_3 | 0.01 | Q60_1 | 0.01 | Q18_3 | 0.01 | A25_2 | 0.009 |
| Q11_3 | 0.009 | Q17_0 | 0.01 | A25_2 | 0.01 | Q17_0 | 0.009 | Q42_4 | 0.009 |
| Q23_0 | 0.009 | A32_4 | 0.009 | Q11_3 | 0.009 | A25_2 | 0.009 | age_10 | 0.009 |
| Q18_3 | 0.009 | Q11_3 | 0.009 | age_10 | 0.009 | Q11_3 | 0.009 | A32_4 | 0.009 |
| Q2_2 | 0.009 | age_10 | 0.009 | Q6_4 | 0.009 | age_10 | 0.009 | Q11_3 | 0.009 |
| age_10 | 0.009 | Q42_4 | 0.009 | Q42_4 | 0.009 | A32_4 | 0.009 | Q6_4 | 0.009 |
| Q12_2 | 0.008 | Q6_4 | 0.009 | A32_4 | 0.009 | Q42_4 | 0.009 | Q41_0 | 0.008 |
| Q42_4 | 0.008 | A5_0 | 0.009 | A5_1 | 0.008 | Q6_4 | 0.009 | Q1_4 | 0.008 |
| Q1_4 | 0.008 | Q1_4 | 0.008 | Q1_4 | 0.008 | A5_1 | 0.008 | A32_3 | 0.008 |
| Q13_0 | 0.008 | A32_3 | 0.008 | A32_3 | 0.008 | Q1_4 | 0.008 | A5_1 | 0.008 |
| B1_3 | 0.008 | Q60_2 | 0.008 | Q73_1 | 0.008 | A32_3 | 0.008 | Q60_2 | 0.008 |
| Q22_0 | 0.008 | Q73_0 | 0.008 | Q60_2 | 0.007 | Q60_2 | 0.008 | Q73_0 | 0.008 |
| A32_3 | 0.008 | Q34_0 | 0.007 | Q1_1 | 0.007 | Q73_1 | 0.008 | Q1_1 | 0.007 |
| A32_4 | 0.008 | Q1_1 | 0.007 | Q68_1 | 0.007 | Q1_1 | 0.007 | B1_3 | 0.007 |
| A5_1 | 0.008 | Q68_0 | 0.007 | B1_3 | 0.007 | Q68_1 | 0.007 | Q34_0 | 0.007 |
| Q23_1 | 0.007 | B1_3 | 0.007 | Q25_1 | 0.007 | B1_3 | 0.007 | Q68_0 | 0.007 |
| Q60_2 | 0.007 | Q21_5 | 0.007 | Q34_0 | 0.007 | Q34_0 | 0.007 | Q21_5 | 0.006 |
| Q34_1 | 0.007 | Q25_1 | 0.006 | Q21_5 | 0.006 | Q21_5 | 0.006 | Q2_1 | 0.006 |
| Q1_1 | 0.007 | Q30_0 | 0.006 | Q30_0 | 0.006 | Q25_1 | 0.006 | Q30_0 | 0.006 |
| Q21_5 | 0.007 | age_9 | 0.006 | age_9 | 0.006 | Q30_0 | 0.006 | Q25_1 | 0.006 |
| Q8_1 | 0.006 | Q8_1 | 0.006 | Q3_3 | 0.006 | age_9 | 0.006 | age_9 | 0.006 |
| Q25_1 | 0.006 | Q42_0 | 0.006 | Q8_1 | 0.006 | Q3_3 | 0.006 | Q8_1 | 0.006 |
| Q68_0 | 0.006 | Q31_2 | 0.005 | Q31_2 | 0.005 | Q8_1 | 0.006 | Q40_0 | 0.005 |
| Q12_3 | 0.006 | Q3_3 | 0.005 | Q67_1 | 0.005 | Q42_0 | 0.005 | Q67_1 | 0.005 |
| Q3_3 | 0.006 | Q13_2 | 0.005 | Q42_0 | 0.005 | Q67_1 | 0.005 | Q31_2 | 0.005 |
| Q67_1 | 0.006 | Q42_1 | 0.005 | Q2_1 | 0.005 | Q31_2 | 0.005 | Q3_3 | 0.005 |
| Q30_0 | 0.006 | Q67_1 | 0.005 | Q42_1 | 0.005 | Q13_2 | 0.005 | Q13_2 | 0.005 |
| age_9 | 0.005 | Q18_4 | 0.005 | Q13_2 | 0.005 | Q2_1 | 0.005 | Q18_4 | 0.005 |
| B1_0 | 0.005 | Q2_1 | 0.005 | Q18_4 | 0.005 | Q18_4 | 0.005 | Q11_4 | 0.005 |
| Q18_2 | 0.005 | Q11_4 | 0.005 | Q11_4 | 0.004 | Q42_1 | 0.005 | Q13_3 | 0.004 |
| Q31_2 | 0.005 | Q13_3 | 0.004 | Q13_3 | 0.004 | Q11_4 | 0.005 | Q42_1 | 0.004 |
| Q13_2 | 0.005 | Q40_5 | 0.004 | Q18_2 | 0.004 | Q13_3 | 0.004 | Q18_2 | 0.004 |
| Q42_1 | 0.004 | Q18_2 | 0.004 | A32_1 | 0.004 | Q18_2 | 0.004 | A32_1 | 0.004 |
| Q11_4 | 0.004 | A32_1 | 0.004 | A25_0 | 0.004 | A32_1 | 0.004 | Q31_0 | 0.004 |
| Q2_1 | 0.004 | Q21_0 | 0.004 | Q40_5 | 0.004 | Q40_5 | 0.004 | B1_0 | 0.004 |
| Q40_5 | 0.004 | Q31_0 | 0.004 | Q31_0 | 0.004 | B1_0 | 0.004 | Q40_5 | 0.004 |
| A32_1 | 0.004 | Q53_0 | 0.004 | B1_0 | 0.004 | Q31_0 | 0.004 | Q3_2 | 0.004 |
| pattern_of_infant_feeding_2 | 0.004 | Q8_3 | 0.004 | Q8_3 | 0.004 | A25_0 | 0.004 | Q8_3 | 0.004 |
| Q3_2 | 0.004 | B1_0 | 0.004 | Q10_2 | 0.003 | Q8_3 | 0.004 | A25_0 | 0.003 |
| Q18_4 | 0.004 | Q3_2 | 0.003 | Q33_0 | 0.003 | Q40_3 | 0.003 | Q33_0 | 0.003 |
| Q38_1 | 0.004 | Q40_3 | 0.003 | Q3_2 | 0.003 | Q3_2 | 0.003 | Q10_2 | 0.003 |
| Q8_3 | 0.004 | Q10_2 | 0.003 | Q40_3 | 0.003 | Q10_2 | 0.003 | Q47_0 | 0.003 |
| Q13_3 | 0.003 | Q17_1 | 0.003 | Q47_1 | 0.003 | Q33_1 | 0.003 | B1_2 | 0.003 |
| Q53_0 | 0.003 | Q47_1 | 0.003 | B1_2 | 0.003 | Q47_0 | 0.003 | Q17_1 | 0.003 |
| Q40_3 | 0.003 | B1_2 | 0.003 | Q53_1 | 0.002 | Q17_1 | 0.003 | Q53_1 | 0.002 |
| Q31_0 | 0.003 | Q33_0 | 0.002 | A7_2 | 0.002 | B1_2 | 0.003 | Q48_0 | 0.002 |
| Q10_2 | 0.003 | A7_2 | 0.002 | Q21_0 | 0.002 | Q53_1 | 0.002 | Q40_3 | 0.002 |
| B1_2 | 0.003 | C8_0 | 0.002 | Q48_0 | 0.002 | A7_2 | 0.002 | C8_0 | 0.002 |
| Q47_0 | 0.002 | B9_0 | 0.002 | C8_0 | 0.002 | Q6_0 | 0.002 | A7_2 | 0.002 |
| A7_2 | 0.002 | Q38_1 | 0.002 | Q6_0 | 0.002 | B9_1 | 0.002 | B9_0 | 0.002 |
| Q6_0 | 0.002 | Q48_1 | 0.002 | B9_0 | 0.002 | Q48_1 | 0.002 | Q19_2 | 0.002 |
| age_11 | 0.002 | Q6_0 | 0.002 | Hip/height_ratio | 0.002 | C8_1 | 0.002 | Q38_1 | 0.002 |
| A25_0 | 0.002 | Q19_2 | 0.002 | Q38_1 | 0.001 | Q19_2 | 0.001 | Q44_6 | 0.002 |
| Q45_2 | 0.002 | age_11 | 0.001 | age_11 | 0.001 | Q38_0 | 0.001 | age_11 | 0.002 |
| Q35_1 | 0.002 | Q40_0 | 0.001 | Q44_6 | 0.001 | age_11 | 0.001 | Q6_0 | 0.001 |
| Q33_1 | 0.001 | Q1_2 | 0.001 | Q1_2 | 0.001 | Q40_0 | 0.001 | Q1_2 | 0.001 |
| Q48_1 | 0.001 | Q45_2 | 0.001 | Q45_5 | 0.001 | Q21_0 | 0.001 | Q21_0 | 0.001 |
| Q67_0 | 0.001 | Q44_6 | 0.001 | Q45_2 | 0.001 | Q44_6 | 0.001 | Q3_4 | 0.001 |
| Q19_2 | 0.001 | Q45_5 | 0.001 | Q19_2 | 0.001 | Q45_2 | 0.001 | Q45_5 | 0.001 |
| Q28_0 | 0.001 | Q3_4 | 0.001 | Q3_4 | 0.001 | Q1_2 | 0.001 | Q45_2 | 0.001 |
| B9_1 | 0.001 | Q28_0 | 0.001 | Q28_0 | 0.001 | Q45_5 | 0.001 | Hip/height_ratio | 0.001 |
| Q21_0 | 0.001 | Q52_1 | 0.001 | Q52_1 | 0.001 | Q3_4 | 0.001 | Q28_0 | 0.001 |
| Q44_6 | 0.001 | Q26_0 | 0 | Waist_circumference | 0.001 | Q52_0 | 0.001 | Q52_0 | 0.001 |
| Q45_5 | 0.001 | B1_1 | 0 | Q26_0 | 0 | Q28_0 | 0.001 | Q26_0 | 0 |
| Q52_1 | 0.001 | Q1_3 | 0 | Q1_3 | 0 | Q60_0 | 0 | Q60_0 | 0 |
| C8_1 | 0.001 | Q60_0 | 0 | Q35_0 | 0 | Q26_0 | 0 | Q10_1 | 0 |
| Hip/height_ratio | 0 | age_13 | 0 | Q67_4 | 0 | Hip/height_ratio | 0 | Q42_3 | 0 |
| Q26_0 | 0 | age_14 | 0 | Q44_5 | 0 | Q1_3 | 0 | Q1_3 | 0 |
| Q1_2 | 0 | Q44_1 | 0 | Q44_2 | 0 | age_12 | 0 | Q53_ | 0 |
| Q3_4 | 0 | age_4 | 0 | age_12 | 0 | age_13 | 0 | Born_at_term_ | 0 |
| Q28_2 | 0 | Q44_2 | 0 | Q49_0 | 0 | Q42_2 | 0 | age_8 | 0 |
| Q45_4 | 0 | age_8 | 0 | Q74_0 | 0 | Q67_0 | 0 | Q49_0 | 0 |
| C5_1 | 0 | Q42_6 | 0 | Q60_0 | 0 | Q60_ | 0 | Waist_circumference | 0 |
| age_8 | 0 | Q60_4 | 0 | Q60_5 | 0 | Q67_2 | 0 | B1_4 | 0 |
| A25_1 | 0 | age_12 | 0 | Q67_0 | 0 | age_8 | 0 | B6_0 | 0 |
| Born_at_term_ | 0 | Q44_5 | 0 | Q67_3 | 0 | Q42_3 | 0 | A3_3 | 0 |
| Waist_circumference | 0 | Q45_4 | 0 | age_4 | 0 | Q49_0 | 0 | A24_ | 0 |
| Q74_0 | 0 | Q74_0 | 0 | Q67_2 | 0 | Q67_3 | 0 | A3_5 | 0 |
| Q49_0 | 0 | Q67_5 | 0 | age_13 | 0 | Q67_4 | 0 | Q74_1 | 0 |
| age_13 | 0 | Q67_4 | 0 | age_14 | 0 | Q67_5 | 0 | A7_3 | 0 |
| A7_3 | 0 | Q42_5 | 0 | Q67_5 | 0 | Q42_5 | 0 | A25_1 | 0 |
| Q67_5 | 0 | Q67_2 | 0 | age_17 | 0 | age_4 | 0 | C5_1 | 0 |
| Q67_4 | 0 | Q49_0 | 0 | age_8 | 0 | Q45_4 | 0 | age_12 | 0 |
| Q67_3 | 0 | Q67_0 | 0 | age_15 | 0 | Q74_1 | 0 | Q67_5 | 0 |
| age_12 | 0 | Q60_5 | 0 | Q45_4 | 0 | Q45_1 | 0 | Q67_3 | 0 |
| Q67_2 | 0 | Q67_3 | 0 | Q44_1 | 0 | Q42_6 | 0 | Q67_2 | 0 |
| Q44_5 | 0 | BMI | 0 | BMI | 0 | Q44_5 | 0 | age_13 | 0 |
| age_4 | 0 | Q42_3 | 0 | Q42_6 | 0 | Q60_4 | 0 | age_14 | 0 |
| A3_3 | 0 | Q42_2 | 0 | Q42_5 | 0 | BMI | 0 | Q67_0 | 0 |
| Q67_ | 0 | Q8_0 | 0 | Q10_1 | 0 | Q40_6 | 0 | Q67_ | 0 |
| Q60_4 | 0 | Q8_ | 0 | Q8_0 | 0 | B6_0 | 0 | age_4 | 0 |
| A24_ | 0 | Q7_4 | 0 | Q8_ | 0 | Q10_3 | 0 | Q60_4 | 0 |
| A4_ | 0 | Q7_3 | 0 | Q7_4 | 0 | Q10_1 | 0 | A6_ | 0 |
| Q60_0 | 0 | Q7_1 | 0 | Q7_3 | 0 | Q8_0 | 0 | B1_1 | 0 |
| A6_ | 0 | Q6_3 | 0 | Q7_1 | 0 | Q8_ | 0 | Q67_4 | 0 |
| A3_5 | 0 | Q6_2 | 0 | Q6_3 | 0 | Q7_4 | 0 | Q42_2 | 0 |
| Q8_0 | 0 | Q6_1 | 0 | Q6_2 | 0 | Q7_3 | 0 | Q45_4 | 0 |
| Q32_ | 0 | Q3_1 | 0 | Q6_1 | 0 | Q7_1 | 0 | Q21_4 | 0 |
| C10_ | 0 | B6_1 | 0 | Q3_1 | 0 | Q6_3 | 0 | Q8_0 | 0 |
| Q13_4 | 0 | B1_4 | 0 | B6_1 | 0 | Q6_2 | 0 | Q26_1 | 0 |
| Q1_3 | 0 | C18_ | 0 | B1_4 | 0 | Q6_1 | 0 | Q10_3 | 0 |
| Q19_5 | 0 | C5_1 | 0 | B1_1 | 0 | Q3_1 | 0 | Q24_2 | 0 |
| Q19_4 | 0 | C3_ | 0 | C10_ | 0 | B1_4 | 0 | Q24_ | 0 |
| Q19_1 | 0 | A25_1 | 0 | C5_1 | 0 | Q40_1 | 0 | Q13_1 | 0 |
| Q19_ | 0 | A24_ | 0 | A25_1 | 0 | B1_1 | 0 | Q22_ | 0 |
| Q18_5 | 0 | A7_3 | 0 | A24_ | 0 | C10_ | 0 | Q13_4 | 0 |
| Q3_1 | 0 | A6_ | 0 | A7_3 | 0 | C5_0 | 0 | Q21_3 | 0 |
| Q18_0 | 0 | A4_ | 0 | A6_ | 0 | A25_1 | 0 | Q44_5 | 0 |
| Q18_ | 0 | A3_5 | 0 | A4_ | 0 | A7_3 | 0 | Q21_2 | 0 |
| Q13_1 | 0 | A3_3 | 0 | A3_5 | 0 | A4_ | 0 | Q17_ | 0 |
| Q44_2 | 0 | Hip/height_ratio | 0 | A3_3 | 0 | A3_5 | 0 | Q21_ | 0 |
| Q12_1 | 0 | Waist_circumference | 0 | A3_ | 0 | A3_3 | 0 | Q19_4 | 0 |
| Q6_1 | 0 | Q10_1 | 0 | Q10_3 | 0 | A3_ | 0 | Q19_1 | 0 |
| Q6_2 | 0 | Q13_1 | 0 | Q13_1 | 0 | A2_ | 0 | Q17_2 | 0 |
| Q6_3 | 0 | Q17_2 | 0 | Q13_4 | 0 | Waist_circumference | 0 | Q18_5 | 0 |
| Q10_3 | 0 | Q26_1 | 0 | Q26_1 | 0 | Q13_1 | 0 | Q17_3 | 0 |
| Q10_1 | 0 | Q40_6 | 0 | Q42_3 | 0 | Q13_4 | 0 | Q27_2 | 0 |
| Q7_1 | 0 | Q40_1 | 0 | Q42_2 | 0 | Q17_2 | 0 | Q28_1 | 0 |
| Q7_3 | 0 | Q37_0 | 0 | Q40_6 | 0 | Q17_3 | 0 | Q28_2 | 0 |
| Q7_4 | 0 | Q35_1 | 0 | Q40_1 | 0 | Q37_0 | 0 | Q8_ | 0 |
| Q21_2 | 0 | Q32_ | 0 | Q37_1 | 0 | Q35_1 | 0 | Q44_2 | 0 |
| Q21_3 | 0 | Q31_1 | 0 | Q31_1 | 0 | Q31_1 | 0 | Q42_6 | 0 |
| Q21_4 | 0 | Q30_2 | 0 | Q30_2 | 0 | Q31_ | 0 | Q42_5 | 0 |
| Q22_ | 0 | Q28_2 | 0 | Q28_2 | 0 | Q30_2 | 0 | Q3_1 | 0 |
| Q44_1 | 0 | Q28_1 | 0 | Q28_1 | 0 | Q28_2 | 0 | Q18_0 | 0 |
| Q42_6 | 0 | Q27_ | 0 | Q27_2 | 0 | Q28_1 | 0 | Q40_6 | 0 |
| Q42_5 | 0 | Q24_2 | 0 | Q24_2 | 0 | Q28_ | 0 | Q40_1 | 0 |
| Q42_3 | 0 | Q17_3 | 0 | Q18_0 | 0 | Q27_2 | 0 | Q6_1 | 0 |
| Q42_2 | 0 | Q24_ | 0 | Q24_ | 0 | Q26_1 | 0 | Q37_0 | 0 |
| Q40_6 | 0 | Q21_4 | 0 | Q22_ | 0 | Q26_ | 0 | Q6_2 | 0 |
| Q40_1 | 0 | Q21_3 | 0 | Q21_4 | 0 | Q24_2 | 0 | Q35_1 | 0 |
| Q37_1 | 0 | Q21_2 | 0 | Q21_3 | 0 | Q22_ | 0 | Q6_3 | 0 |
| B1_1 | 0 | Q19_5 | 0 | Q21_2 | 0 | Q21_4 | 0 | Q7_1 | 0 |
| Q31_1 | 0 | Q19_4 | 0 | Q21_ | 0 | Q21_3 | 0 | Q7_3 | 0 |
| Q30_2 | 0 | Q19_1 | 0 | Q19_5 | 0 | Q21_2 | 0 | Q31_1 | 0 |
| Q28_1 | 0 | Q18_5 | 0 | Q19_4 | 0 | Q19_5 | 0 | Q30_2 | 0 |
| Q28_ | 0 | Q18_0 | 0 | Q19_1 | 0 | Q19_4 | 0 | Q7_4 | 0 |
| Q27_2 | 0 | Q17_5 | 0 | Q18_5 | 0 | Q19_1 | 0 | BMI | 0 |
| B1_4 | 0 | pattern_of_infant_feeding_ | 0 | pattern_of_infant_feeding_ | 0 | Q18_5 | 0 |  |  |
| B6_1 | 0 |  |  |  |  | Q18_0 | 0 |  |  |
| Q26_1 | 0 |  |  |  |  | Q18_ | 0 |  |  |
| Q24_2 | 0 |  |  |  |  | Q17_5 | 0 |  |  |
| Q24_ | 0 |  |  |  |  | pattern_of_infant_feeding_ | 0 |  |  |
| BMI | 0 |  |  |  |  |  |  |  |  |

| AHI≥10 as the cut-off value | | | | | | | | | |
| --- | --- | --- | --- | --- | --- | --- | --- | --- | --- |
| Random Seed=786 | | Random Seed=256 | | Random Seed=321 | | Random Seed=423 | | Random Seed=521 | |
| Variable | Value | Variable | Value | Variable | Value | Variable | Value | Variable | Value |
| A7_4 | 0.096 | A7_4 | 0.098 | A7_4 | 0.097 | A7_4 | 0.098 | A7_4 | 0.098 |
| A2_1 | 0.076 | A2_0 | 0.071 | A2_0 | 0.071 | A2_0 | 0.071 | A6_0 | 0.072 |
| A6_1 | 0.068 | A6_1 | 0.070 | A6_1 | 0.069 | A6_1 | 0.070 | A2_0 | 0.070 |
| Q12_0 | 0.065 | A3_4 | 0.057 | A3_4 | 0.056 | A3_4 | 0.056 | A3_4 | 0.058 |
| A3_4 | 0.056 | Q4_4 | 0.056 | Q4_4 | 0.056 | Q4_4 | 0.056 | Q4_4 | 0.056 |
| Hip_circumference | 0.054 | Hip_circumference | 0.053 | Hip_circumference | 0.053 | Hip_circumference | 0.052 | Hip_circumference | 0.052 |
| Q4_4 | 0.054 | A4_1 | 0.052 | A4_1 | 0.051 | A4_1 | 0.051 | A4_0 | 0.049 |
| A4_0 | 0.046 | A7_0 | 0.045 | A7_0 | 0.044 | A7_0 | 0.045 | A7_0 | 0.044 |
| A7_0 | 0.044 | Q45_3 | 0.037 | A24_1 | 0.038 | A24_1 | 0.038 | Q45_3 | 0.037 |
| A24_1 | 0.040 | A24_0 | 0.036 | Q45_3 | 0.036 | Q45_3 | 0.036 | A24_0 | 0.036 |
| Q27_0 | 0.035 | Q27_0 | 0.034 | Neck/height_ratio | 0.034 | Q27_0 | 0.034 | Neck/height_ratio | 0.033 |
| Q45_3 | 0.035 | Neck/height_ratio | 0.033 | Q27_0 | 0.033 | Neck/height_ratio | 0.033 | C14_1 | 0.033 |
| Neck/height_ratio | 0.032 | C14_1 | 0.033 | Waist/hip_ratio | 0.032 | Waist/hip_ratio | 0.032 | Waist/hip_ratio | 0.031 |
| Waist/hip_ratio | 0.032 | Waist/hip_ratio | 0.032 | C14_0 | 0.031 | C14_1 | 0.031 | sex_1 | 0.029 |
| C14_1 | 0.030 | sex_2 | 0.029 | sex_1 | 0.029 | sex_2 | 0.029 | B22_0 | 0.026 |
| sex_1 | 0.029 | C3_0 | 0.025 | C3_1 | 0.025 | B22_1 | 0.024 | C3_0 | 0.025 |
| C3_0 | 0.025 | B22_0 | 0.025 | B22_1 | 0.025 | C3_0 | 0.024 | Q27_1 | 0.024 |
| B22_1 | 0.022 | Q30_0 | 0.020 | Q30_0 | 0.019 | Q30_0 | 0.020 | Q26_2 | 0.021 |
| Q3_0 | 0.019 | Q19_0 | 0.017 | Q19_0 | 0.016 | Q19_0 | 0.016 | Q3_0 | 0.018 |
| Q30_0 | 0.018 | Q3_0 | 0.016 | Q3_0 | 0.015 | Q3_0 | 0.015 | Q30_0 | 0.017 |
| Q19_0 | 0.018 | Q26_2 | 0.016 | Q26_2 | 0.015 | Q26_2 | 0.015 | Q19_0 | 0.016 |
| Q10_0 | 0.017 | Q10_0 | 0.015 | Q10_0 | 0.015 | Q10_0 | 0.015 | Q10_0 | 0.016 |
| Q26_2 | 0.015 | C10_0 | 0.011 | C10_0 | 0.012 | C10_1 | 0.014 | C10_0 | 0.012 |
| C10_1 | 0.014 | C5_1 | 0.011 | Q6_0 | 0.010 | Q6_0 | 0.010 | Q7_0 | 0.011 |
| Q6_0 | 0.010 | Q7_0 | 0.010 | Q7_0 | 0.010 | C5_0 | 0.010 | C5_1 | 0.011 |
| Q7_0 | 0.010 | Q6_0 | 0.010 | C5_1 | 0.010 | Q7_0 | 0.010 | Q6_0 | 0.011 |
| Neck_circumference | 0.010 | Q73_0 | 0.009 | Neck_circumference | 0.008 | Neck_circumference | 0.009 | Q73_0 | 0.010 |
| C5_1 | 0.008 | Neck_circumference | 0.008 | Q73_1 | 0.008 | Q73_1 | 0.008 | Neck_circumference | 0.008 |
| Q6_4 | 0.007 | Q69_1 | 0.008 | Q69_1 | 0.007 | Q69_0 | 0.007 | Q69_1 | 0.007 |
| Q69_0 | 0.006 | A25_0 | 0.007 | A25_0 | 0.006 | A25_0 | 0.007 | Q6_4 | 0.006 |
| Q45_5 | 0.004 | Q6_4 | 0.006 | Q6_4 | 0.005 | Q6_4 | 0.005 | A25_0 | 0.006 |
| Q23_1 | 0.002 | Q45_5 | 0.005 | Q45_5 | 0.005 | Q45_5 | 0.005 | Q45_5 | 0.005 |
| A25_0 | 0.001 | Q23_1 | 0.003 | Q2_4 | 0.002 | Q2_4 | 0.002 | Q2_4 | 0.003 |
| Q72_1 | 0.000 | Q2_4 | 0.002 | Q23_0 | 0.001 | pattern_of_infant_feeding_1 | 0.002 | Q23_1 | 0.003 |
| Q2_4 | 0.000 | pattern_of_infant_feeding_1 | 0.002 | Q72_1 | 0.001 | Q72_0 | 0.001 | Q40_0 | 0.003 |
| Q28_0 | 0.000 | Q72_1 | 0.002 | Q31_1 | 0.000 | Q23_0 | 0.001 | Q72_1 | 0.001 |
| Q40_1 | 0.000 | age_10 | 0.000 | Q31_2 | 0.000 | Q68_1 | 0.000 | Q40_5 | 0.000 |
| Q24_2 | 0.000 | Q38_1 | 0.000 | Q32_ | 0.000 | Q36_0 | 0.000 | Q25_ | 0.000 |
| Q40_2 | 0.000 | Q31_2 | 0.000 | Q32_1 | 0.000 | Q31_0 | 0.000 | Q24_3 | 0.000 |
| Q24_1 | 0.000 | Q32_ | 0.000 | Q33_0 | 0.000 | Q31_1 | 0.000 | Q40_3 | 0.000 |
| Q40_3 | 0.000 | Q32_0 | 0.000 | Q34_0 | 0.000 | Q31_2 | 0.000 | Q24_2 | 0.000 |
| Q40_4 | 0.000 | Q33_0 | 0.000 | Q35_0 | 0.000 | Q32_ | 0.000 | Q40_4 | 0.000 |
| Q40_5 | 0.000 | Q34_0 | 0.000 | Q36_1 | 0.000 | Q32_1 | 0.000 | Q40_6 | 0.000 |
| Q24_ | 0.000 | Q35_1 | 0.000 | Q37_1 | 0.000 | Q33_1 | 0.000 | Q40_1 | 0.000 |
| Q40_6 | 0.000 | Q36_0 | 0.000 | Q38_1 | 0.000 | Q34_0 | 0.000 | Q41_0 | 0.000 |
| Q42_1 | 0.000 | Q37_0 | 0.000 | Q70_0 | 0.000 | Q35_1 | 0.000 | Q24_1 | 0.000 |
| Q42_2 | 0.000 | Q40_1 | 0.000 | Q40_2 | 0.000 | Q37_0 | 0.000 | Q28_1 | 0.000 |
| Q42_3 | 0.000 | Q42_3 | 0.000 | Q40_3 | 0.000 | Q30_2 | 0.000 | Q42_2 | 0.000 |
| Q42_4 | 0.000 | Q40_2 | 0.000 | age_5 | 0.000 | age_6 | 0.000 | Q42_3 | 0.000 |
| Q27_1 | 0.000 | Q40_3 | 0.000 | Q40_4 | 0.000 | Q38_0 | 0.000 | Q42_4 | 0.000 |
| Q22_0 | 0.000 | Q40_4 | 0.000 | Q40_5 | 0.000 | Q40_1 | 0.000 | Q28_0 | 0.000 |
| Q42_5 | 0.000 | age_6 | 0.000 | Q40_6 | 0.000 | Q40_2 | 0.000 | Q40_2 | 0.000 |
| Q38_1 | 0.000 | Q40_5 | 0.000 | Q42_0 | 0.000 | Q40_3 | 0.000 | Q26_1 | 0.000 |
| Q37_0 | 0.000 | Q40_6 | 0.000 | Q42_1 | 0.000 | Q40_4 | 0.000 | Q26_0 | 0.000 |
| Q36_0 | 0.000 | Q42_0 | 0.000 | Q42_2 | 0.000 | Q40_5 | 0.000 | Q32_ | 0.000 |
| Q30_2 | 0.000 | Q42_1 | 0.000 | Q40_1 | 0.000 | Q31_ | 0.000 | Q28_2 | 0.000 |
| Q26_1 | 0.000 | Q31_1 | 0.000 | Q31_0 | 0.000 | age_7 | 0.000 | Q42_5 | 0.000 |
| Q27_2 | 0.000 | Q31_0 | 0.000 | Q30_2 | 0.000 | Q30_1 | 0.000 | Q30_1 | 0.000 |
| Q28_1 | 0.000 | Q30_2 | 0.000 | Q30_1 | 0.000 | Q42_0 | 0.000 | Q30_2 | 0.000 |
| Q28_2 | 0.000 | Q30_1 | 0.000 | Q24_2 | 0.000 | Q21_5 | 0.000 | Q31_0 | 0.000 |
| Q30_ | 0.000 | Q24_ | 0.000 | Q24_3 | 0.000 | Q22_1 | 0.000 | Q31_1 | 0.000 |
| Q26_0 | 0.000 | Q24_1 | 0.000 | Q25_1 | 0.000 | Q24_1 | 0.000 | Q31_2 | 0.000 |
| Q30_1 | 0.000 | Q24_2 | 0.000 | Q26_ | 0.000 | Q24_2 | 0.000 | Q32_1 | 0.000 |
| Q31_0 | 0.000 | Q24_3 | 0.000 | Q26_0 | 0.000 | Q24_3 | 0.000 | Q38_1 | 0.000 |
| Q35_1 | 0.000 | Q25_1 | 0.000 | Q26_1 | 0.000 | pattern_of_infant_feeding_ | 0.000 | Q33_0 | 0.000 |
| Q31_1 | 0.000 | Q26_0 | 0.000 | Born_at_term_ | 0.000 | Q25_1 | 0.000 | Q34_0 | 0.000 |
| Q25_1 | 0.000 | Q26_1 | 0.000 | age_9 | 0.000 | Q26_0 | 0.000 | Q34_1 | 0.000 |
| Q31_2 | 0.000 | Born_at_term_0 | 0.000 | Q27_1 | 0.000 | Q26_1 | 0.000 | Q25_1 | 0.000 |
| Q32_0 | 0.000 | Born_at_term_ | 0.000 | Q27_2 | 0.000 | Born_at_term_1 | 0.000 | Q27_2 | 0.000 |
| Q33_1 | 0.000 | age_9 | 0.000 | Q28_ | 0.000 | age_9 | 0.000 | Q36_0 | 0.000 |
| Q27_ | 0.000 | age_8 | 0.000 | age_8 | 0.000 | age_8 | 0.000 | Q37_0 | 0.000 |
| Q24_3 | 0.000 | Q27_1 | 0.000 | age_7 | 0.000 | Q27_1 | 0.000 | Q35_1 | 0.000 |
| Q34_1 | 0.000 | Q27_2 | 0.000 | Q28_0 | 0.000 | Q27_2 | 0.000 | BMI | 0.000 |
| BMI | 0.000 | Q28_0 | 0.000 | Q28_1 | 0.000 | Q28_0 | 0.000 | Q42_6 | 0.000 |
| Q42_6 | 0.000 | Q28_1 | 0.000 | Q28_2 | 0.000 | Q28_1 | 0.000 | Q44_1 | 0.000 |
| Q67_1 | 0.000 | Q28_2 | 0.000 | age_6 | 0.000 | Q28_2 | 0.000 | Q67_3 | 0.000 |
| Q67_3 | 0.000 | age_7 | 0.000 | Q42_3 | 0.000 | Q40_6 | 0.000 | Q67_4 | 0.000 |
| Q67_4 | 0.000 | Q42_2 | 0.000 | Q42_4 | 0.000 | Q42_1 | 0.000 | Q67_5 | 0.000 |
| Q67_5 | 0.000 | Q42_4 | 0.000 | Q42_5 | 0.000 | Q67_5 | 0.000 | Q68_0 | 0.000 |
| Q68_0 | 0.000 | age_11 | 0.000 | Q60_4 | 0.000 | Q45_6 | 0.000 | Q70_1 | 0.000 |
| Q70_1 | 0.000 | Q60_3 | 0.000 | Q49_0 | 0.000 | Q47_0 | 0.000 | Q74_1 | 0.000 |
| Q74_0 | 0.000 | Q48_1 | 0.000 | Q52_1 | 0.000 | Q48_1 | 0.000 | age_10 | 0.000 |
| age_10 | 0.000 | Q49_0 | 0.000 | Q53_1 | 0.000 | Q74_1 | 0.000 | age_11 | 0.000 |
| age_11 | 0.000 | Q52_1 | 0.000 | Q60_0 | 0.000 | Q49_0 | 0.000 | age_12 | 0.000 |
| age_12 | 0.000 | Q53_0 | 0.000 | age_10 | 0.000 | Q52_0 | 0.000 | age_13 | 0.000 |
| age_13 | 0.000 | Q60_0 | 0.000 | Q60_1 | 0.000 | Q53_1 | 0.000 | age_14 | 0.000 |
| age_14 | 0.000 | age_12 | 0.000 | Q60_2 | 0.000 | Q60_0 | 0.000 | age_3 | 0.000 |
| age_3 | 0.000 | Q60_1 | 0.000 | Q60_3 | 0.000 | Q60_1 | 0.000 | age_4 | 0.000 |
| age_4 | 0.000 | Q60_2 | 0.000 | Q60_5 | 0.000 | Q60_2 | 0.000 | age_5 | 0.000 |
| age_5 | 0.000 | Q60_4 | 0.000 | Q42_6 | 0.000 | Q60_3 | 0.000 | age_6 | 0.000 |
| age_6 | 0.000 | Q70_0 | 0.000 | Q67_0 | 0.000 | Q60_4 | 0.000 | age_7 | 0.000 |
| age_7 | 0.000 | Q60_5 | 0.000 | Q67_1 | 0.000 | Q67_0 | 0.000 | age_8 | 0.000 |
| age_8 | 0.000 | Q67_0 | 0.000 | Q67_2 | 0.000 | Q67_1 | 0.000 | age_9 | 0.000 |
| age_9 | 0.000 | Q67_1 | 0.000 | Q67_3 | 0.000 | Q67_2 | 0.000 | Born_at_term_0 | 0.000 |
| Born_at_term_0 | 0.000 | Q67_2 | 0.000 | Q67_4 | 0.000 | Q67_3 | 0.000 | Q67_2 | 0.000 |
| pattern_of_infant_feeding_ | 0.000 | Q67_3 | 0.000 | Q67_5 | 0.000 | Q67_4 | 0.000 | Q67_1 | 0.000 |
| pattern_of_infant_feeding_2 | 0.000 | Q67_4 | 0.000 | Q68_1 | 0.000 | Q70_0 | 0.000 | Q67_0 | 0.000 |
| Q67_2 | 0.000 | Q67_5 | 0.000 | Q74_0 | 0.000 | Q46_1 | 0.000 | Q46_0 | 0.000 |
| Q67_0 | 0.000 | Q68_0 | 0.000 | Q48_0 | 0.000 | age_10 | 0.000 | Q44_2 | 0.000 |
| Q44_1 | 0.000 | Q47_1 | 0.000 | Q47_1 | 0.000 | age_5 | 0.000 | Q44_3 | 0.000 |
| Q67_ | 0.000 | Q46_0 | 0.000 | Q46_0 | 0.000 | Q45_4 | 0.000 | Q44_4 | 0.000 |
| Q44_2 | 0.000 | Q45_6 | 0.000 | age_11 | 0.000 | age_4 | 0.000 | Q44_5 | 0.000 |
| Q44_3 | 0.000 | Q74_0 | 0.000 | age_4 | 0.000 | Q42_2 | 0.000 | Q44_6 | 0.000 |
| Q44_4 | 0.000 | Q42_6 | 0.000 | age_3 | 0.000 | Q42_3 | 0.000 | Q45_2 | 0.000 |
| Q44_5 | 0.000 | age_5 | 0.000 | Q43_0 | 0.000 | Q42_4 | 0.000 | Q45_4 | 0.000 |
| Q44_6 | 0.000 | age_4 | 0.000 | Q44_1 | 0.000 | age_3 | 0.000 | Q45_6 | 0.000 |
| Q45_2 | 0.000 | Q43_1 | 0.000 | Q44_2 | 0.000 | age_13 | 0.000 | Q47_0 | 0.000 |
| Q45_4 | 0.000 | Q44_1 | 0.000 | age_15 | 0.000 | Q42_5 | 0.000 | Q60_4 | 0.000 |
| Q21_4 | 0.000 | Q44_2 | 0.000 | age_14 | 0.000 | Q42_6 | 0.000 | Q48_0 | 0.000 |
| Q45_6 | 0.000 | age_3 | 0.000 | Q44_3 | 0.000 | Q43_0 | 0.000 | Q49_0 | 0.000 |
| Q46_1 | 0.000 | age_15 | 0.000 | Q44_4 | 0.000 | Q44_3 | 0.000 | Q52_0 | 0.000 |
| Q47_0 | 0.000 | Q44_3 | 0.000 | Q44_5 | 0.000 | Q44_4 | 0.000 | Q53_1 | 0.000 |
| Q48_1 | 0.000 | Q44_4 | 0.000 | Q44_6 | 0.000 | Q44_5 | 0.000 | Q60_0 | 0.000 |
| Q49_0 | 0.000 | Q44_5 | 0.000 | Q45_2 | 0.000 | Q44_6 | 0.000 | Q60_1 | 0.000 |
| Q52_1 | 0.000 | Q44_6 | 0.000 | age_13 | 0.000 | Q45_2 | 0.000 | Q60_2 | 0.000 |
| Q53_0 | 0.000 | Q45_2 | 0.000 | Q45_4 | 0.000 | age_12 | 0.000 | Q60_3 | 0.000 |
| Q60_0 | 0.000 | age_14 | 0.000 | Q24_1 | 0.000 | Q21_3 | 0.000 | Q24_ | 0.000 |
| Q60_1 | 0.000 | Q45_4 | 0.000 | Q45_6 | 0.000 | age_11 | 0.000 | Q19_1 | 0.000 |
| Q60_2 | 0.000 | age_13 | 0.000 | age_12 | 0.000 | Q21_4 | 0.000 | Q22_1 | 0.000 |
| Q60_3 | 0.000 | Q22_0 | 0.000 | BMI | 0.000 | BMI | 0.000 | Q1_3 | 0.000 |
| Q60_4 | 0.000 | Q42_5 | 0.000 | Q19_1 | 0.000 | Q21_2 | 0.000 | B1_0 | 0.000 |
| Q60_5 | 0.000 | BMI | 0.000 | Q24_ | 0.000 | B9_1 | 0.000 | B1_1 | 0.000 |
| Q21_5 | 0.000 | Q21_5 | 0.000 | Q1_2 | 0.000 | A32_2 | 0.000 | B1_2 | 0.000 |
| Q18_4 | 0.000 | Q21_4 | 0.000 | A32_4 | 0.000 | A32_3 | 0.000 | B1_3 | 0.000 |
| Q21_3 | 0.000 | A32_3 | 0.000 | B1_0 | 0.000 | A32_4 | 0.000 | B1_4 | 0.000 |
| Q21_2 | 0.000 | A32_4 | 0.000 | B1_1 | 0.000 | B1_0 | 0.000 | B6_0 | 0.000 |
| A32_3 | 0.000 | B1_0 | 0.000 | B1_2 | 0.000 | B1_1 | 0.000 | B9_0 | 0.000 |
| A32_4 | 0.000 | B1_1 | 0.000 | B1_3 | 0.000 | B1_2 | 0.000 | Q1_1 | 0.000 |
| B1_0 | 0.000 | B1_2 | 0.000 | B1_4 | 0.000 | B1_3 | 0.000 | Q1_2 | 0.000 |
| B1_1 | 0.000 | B1_3 | 0.000 | B6_1 | 0.000 | B1_4 | 0.000 | Q1_4 | 0.000 |
| B1_2 | 0.000 | B1_4 | 0.000 | B9_0 | 0.000 | B6_0 | 0.000 | Q22_0 | 0.000 |
| B1_3 | 0.000 | B6_1 | 0.000 | Q1_1 | 0.000 | Q1_1 | 0.000 | Q2_1 | 0.000 |
| B1_4 | 0.000 | B9_0 | 0.000 | Q1_3 | 0.000 | A32_0 | 0.000 | Q2_2 | 0.000 |
| B6_1 | 0.000 | Q1_1 | 0.000 | A32_2 | 0.000 | Q1_2 | 0.000 | Q2_3 | 0.000 |
| B9_1 | 0.000 | Q1_2 | 0.000 | Q1_4 | 0.000 | Q1_3 | 0.000 | Q3_1 | 0.000 |
| Q1_1 | 0.000 | Q1_3 | 0.000 | Q2_1 | 0.000 | Q1_4 | 0.000 | Q3_2 | 0.000 |
| Q1_2 | 0.000 | Q1_4 | 0.000 | Q2_2 | 0.000 | Q2_1 | 0.000 | Q3_3 | 0.000 |
| Q1_3 | 0.000 | Q2_ | 0.000 | Q2_3 | 0.000 | Q2_2 | 0.000 | Q3_4 | 0.000 |
| Q1_4 | 0.000 | Q2_1 | 0.000 | Q3_1 | 0.000 | Q2_3 | 0.000 | Q4_0 | 0.000 |
| Q2_1 | 0.000 | Q2_2 | 0.000 | Q3_2 | 0.000 | Q3_1 | 0.000 | Q4_1 | 0.000 |
| Q2_2 | 0.000 | Q2_3 | 0.000 | Q3_3 | 0.000 | Q3_2 | 0.000 | A32_4 | 0.000 |
| Q2_3 | 0.000 | Q3_1 | 0.000 | Q3_4 | 0.000 | Q3_3 | 0.000 | A32_3 | 0.000 |
| Q3_1 | 0.000 | Q3_2 | 0.000 | Q4_ | 0.000 | A32_1 | 0.000 | A32_2 | 0.000 |
| Q3_2 | 0.000 | Q3_3 | 0.000 | A32_3 | 0.000 | C18_1 | 0.000 | A32_1 | 0.000 |
| Q3_3 | 0.000 | Q3_4 | 0.000 | A32_1 | 0.000 | Q21_1 | 0.000 | Waist_circumference | 0.000 |
| Q3_4 | 0.000 | A32_2 | 0.000 | Q22_0 | 0.000 | A4_ | 0.000 | Waist/height_ratio | 0.000 |
| Q4_ | 0.000 | A32_1 | 0.000 | A6_ | 0.000 | Waist_circumference | 0.000 | Hip/height_ratio | 0.000 |
| A32_2 | 0.000 | A32_0 | 0.000 | Waist_circumference | 0.000 | Waist/height_ratio | 0.000 | A2_ | 0.000 |
| A32_1 | 0.000 | A5_0 | 0.000 | Waist/height_ratio | 0.000 | Hip/height_ratio | 0.000 | A3_1 | 0.000 |
| A32_0 | 0.000 | Waist_circumference | 0.000 | Hip/height_ratio | 0.000 | A2_ | 0.000 | A3_2 | 0.000 |
| A5_1 | 0.000 | Waist/height_ratio | 0.000 | A3_0 | 0.000 | A3_0 | 0.000 | A3_3 | 0.000 |
| Waist_circumference | 0.000 | Hip/height_ratio | 0.000 | A3_1 | 0.000 | A3_1 | 0.000 | A3_5 | 0.000 |
| Waist/height_ratio | 0.000 | A2_ | 0.000 | A3_2 | 0.000 | A3_2 | 0.000 | A5_1 | 0.000 |
| Hip/height_ratio | 0.000 | A3_0 | 0.000 | A3_3 | 0.000 | A3_3 | 0.000 | A6_ | 0.000 |
| A3_0 | 0.000 | A3_1 | 0.000 | A3_5 | 0.000 | A3_5 | 0.000 | A7_1 | 0.000 |
| A3_1 | 0.000 | A3_2 | 0.000 | A5_1 | 0.000 | A5_1 | 0.000 | A7_2 | 0.000 |
| A3_2 | 0.000 | A3_3 | 0.000 | A7_1 | 0.000 | C10_ | 0.000 | A7_3 | 0.000 |
| A3_3 | 0.000 | A4_ | 0.000 | A32_0 | 0.000 | A7_1 | 0.000 | A25_2 | 0.000 |
| A3_5 | 0.000 | A6_ | 0.000 | A7_2 | 0.000 | A7_2 | 0.000 | A25_3 | 0.000 |
| A4_ | 0.000 | C18_0 | 0.000 | A7_3 | 0.000 | A7_3 | 0.000 | A25_4 | 0.000 |
| A6_ | 0.000 | A7_1 | 0.000 | A24_ | 0.000 | A24_ | 0.000 | C8_0 | 0.000 |
| C18_1 | 0.000 | A7_2 | 0.000 | A25_1 | 0.000 | A25_1 | 0.000 | C18_0 | 0.000 |
| A7_1 | 0.000 | A7_3 | 0.000 | A25_2 | 0.000 | A25_2 | 0.000 | A32_0 | 0.000 |
| A7_2 | 0.000 | A24_ | 0.000 | A25_3 | 0.000 | A25_3 | 0.000 | Q4_2 | 0.000 |
| A7_3 | 0.000 | A25_1 | 0.000 | A25_4 | 0.000 | A25_4 | 0.000 | Q4_3 | 0.000 |
| A25_1 | 0.000 | A25_2 | 0.000 | C8_0 | 0.000 | C8_1 | 0.000 | Q6_1 | 0.000 |
| A25_2 | 0.000 | A25_3 | 0.000 | C18_0 | 0.000 | Q3_4 | 0.000 | Q13_2 | 0.000 |
| A25_3 | 0.000 | A25_4 | 0.000 | Q4_0 | 0.000 | Q4_0 | 0.000 | Q13_4 | 0.000 |
| A25_4 | 0.000 | C8_0 | 0.000 | Q4_1 | 0.000 | Q4_1 | 0.000 | Q17_0 | 0.000 |
| C8_1 | 0.000 | Q4_0 | 0.000 | Q4_2 | 0.000 | Q18_0 | 0.000 | Q17_1 | 0.000 |
| C10_ | 0.000 | Q4_1 | 0.000 | Q18_5 | 0.000 | Q13_1 | 0.000 | Q18_0 | 0.000 |
| Q4_0 | 0.000 | Q4_2 | 0.000 | Q13_1 | 0.000 | Q13_2 | 0.000 | Q18_1 | 0.000 |
| Q4_1 | 0.000 | Q18_3 | 0.000 | Q13_2 | 0.000 | Q13_3 | 0.000 | Q18_2 | 0.000 |
| Q4_2 | 0.000 | Q13_3 | 0.000 | Q13_3 | 0.000 | Q13_4 | 0.000 | Q18_3 | 0.000 |
| Q18_2 | 0.000 | Q13_4 | 0.000 | Q13_4 | 0.000 | Q17_0 | 0.000 | Q18_4 | 0.000 |
| Q12_3 | 0.000 | Q17_0 | 0.000 | Q18_0 | 0.000 | Q17_1 | 0.000 | Q18_5 | 0.000 |
| Q12_4 | 0.000 | Q17_1 | 0.000 | Q18_1 | 0.000 | Q17_2 | 0.000 | Q19_2 | 0.000 |
| Q13_0 | 0.000 | Q17_2 | 0.000 | Q18_2 | 0.000 | Q17_3 | 0.000 | Q19_3 | 0.000 |
| Q13_1 | 0.000 | Q17_3 | 0.000 | Q18_3 | 0.000 | Q17_4 | 0.000 | Q19_4 | 0.000 |
| Q13_2 | 0.000 | Q18_0 | 0.000 | Q18_4 | 0.000 | Q18_1 | 0.000 | Q21_ | 0.000 |
| Q13_3 | 0.000 | Q18_1 | 0.000 | Q19_2 | 0.000 | Q4_2 | 0.000 | Q21_0 | 0.000 |
| Q13_4 | 0.000 | Q18_2 | 0.000 | Q4_3 | 0.000 | Q18_3 | 0.000 | Q21_1 | 0.000 |
| Q18_0 | 0.000 | Q18_4 | 0.000 | Q19_3 | 0.000 | Q18_4 | 0.000 | Q21_2 | 0.000 |
| Q18_1 | 0.000 | Q13_1 | 0.000 | Q19_4 | 0.000 | Q18_5 | 0.000 | Q21_3 | 0.000 |
| Q18_3 | 0.000 | Q19_1 | 0.000 | Q19_5 | 0.000 | Q19_1 | 0.000 | Q21_4 | 0.000 |
| Q12_1 | 0.000 | Q19_2 | 0.000 | Q21_0 | 0.000 | Q19_2 | 0.000 | Q21_5 | 0.000 |
| Q18_5 | 0.000 | Q19_3 | 0.000 | Q21_1 | 0.000 | Q19_3 | 0.000 | Q13_3 | 0.000 |
| Q19_1 | 0.000 | Q19_4 | 0.000 | Q21_2 | 0.000 | Q19_4 | 0.000 | Q13_1 | 0.000 |
| Q19_2 | 0.000 | Q19_5 | 0.000 | Q21_3 | 0.000 | Q19_5 | 0.000 | Q6_2 | 0.000 |
| Q19_3 | 0.000 | Q21_0 | 0.000 | Q21_4 | 0.000 | Q21_0 | 0.000 | Q13_0 | 0.000 |
| Q19_4 | 0.000 | Q21_1 | 0.000 | Q21_5 | 0.000 | Q13_0 | 0.000 | Q6_3 | 0.000 |
| Q19_5 | 0.000 | Q21_2 | 0.000 | Q13_0 | 0.000 | Q11_4 | 0.000 | Q7_1 | 0.000 |
| Q21_ | 0.000 | Q21_3 | 0.000 | Q11_4 | 0.000 | Q11_3 | 0.000 | Q7_2 | 0.000 |
| Q21_0 | 0.000 | Q13_2 | 0.000 | Q11_3 | 0.000 | Q11_2 | 0.000 | Q7_3 | 0.000 |
| Q21_1 | 0.000 | Q13_0 | 0.000 | Q11_2 | 0.000 | Q4_3 | 0.000 | Q7_4 | 0.000 |
| Q12_2 | 0.000 | Q4_3 | 0.000 | Q6_1 | 0.000 | Q6_1 | 0.000 | Q8_ | 0.000 |
| Q11_4 | 0.000 | Q8_2 | 0.000 | Q6_2 | 0.000 | Q6_2 | 0.000 | Q8_0 | 0.000 |
| Q4_3 | 0.000 | Q6_1 | 0.000 | Q6_3 | 0.000 | Q6_3 | 0.000 | Q8_1 | 0.000 |
| Q8_1 | 0.000 | Q6_2 | 0.000 | Q7_1 | 0.000 | Q7_1 | 0.000 | Q8_2 | 0.000 |
| Q6_1 | 0.000 | Q6_3 | 0.000 | Q7_2 | 0.000 | Q7_2 | 0.000 | Q8_3 | 0.000 |
| Q6_2 | 0.000 | Q7_1 | 0.000 | Q7_3 | 0.000 | Q7_3 | 0.000 | Q8_4 | 0.000 |
| Q6_3 | 0.000 | Q7_2 | 0.000 | Q7_4 | 0.000 | Q7_4 | 0.000 | Q10_1 | 0.000 |
| Q7_1 | 0.000 | Q7_3 | 0.000 | Q8_ | 0.000 | Q8_0 | 0.000 | Q10_2 | 0.000 |
| Q7_2 | 0.000 | Q7_4 | 0.000 | Q8_0 | 0.000 | Q8_1 | 0.000 | Q10_4 | 0.000 |
| Q7_3 | 0.000 | Q8_0 | 0.000 | Q8_1 | 0.000 | Q8_2 | 0.000 | Q11_0 | 0.000 |
| Q7_4 | 0.000 | Q8_1 | 0.000 | Q8_2 | 0.000 | Q8_3 | 0.000 | Q11_1 | 0.000 |
| Q8_ | 0.000 | Q8_3 | 0.000 | Q8_3 | 0.000 | Q8_4 | 0.000 | Q11_2 | 0.000 |
| Q8_0 | 0.000 | Q11_4 | 0.000 | Q8_4 | 0.000 | Q10_1 | 0.000 | Q11_3 | 0.000 |
| Q8_2 | 0.000 | Q8_4 | 0.000 | Q10_1 | 0.000 | Q10_2 | 0.000 | Q11_4 | 0.000 |
| Q11_3 | 0.000 | Q10_1 | 0.000 | Q10_2 | 0.000 | Q10_3 | 0.000 | pattern_of_infant_feeding_ | 0.000 |
| Q8_3 | 0.000 | Q10_2 | 0.000 | Q10_3 | 0.000 | Q10_4 | 0.000 |  |  |
| Q8_4 | 0.000 | Q10_3 | 0.000 | Q10_4 | 0.000 | Q11_0 | 0.000 |  |  |
| Q10_1 | 0.000 | Q10_4 | 0.000 | Q11_0 | 0.000 | Q11_1 | 0.000 |  |  |
| Q10_2 | 0.000 | Q11_0 | 0.000 | Q11_1 | 0.000 | Q18_2 | 0.000 |  |  |
| Q10_3 | 0.000 | Q11_1 | 0.000 | Born_at_term_0 | 0.000 |  |  |  |  |
| Q10_4 | 0.000 | Q11_2 | 0.000 |  |  |  |  |  |  |
| Q11_0 | 0.000 | Q11_3 | 0.000 |  |  |  |  |  |  |
| Q11_1 | 0.000 | Q18_5 | 0.000 |  |  |  |  |  |  |
| Q11_2 | 0.000 |  |  |  |  |  |  |  |  |
| pattern_of_infant_feeding_3 | 0.000 |  |  |  |  |  |  |  |  |
