# Supplementary material for: Pediatric obstructive sleep apnea diagnosis: leveraging machine learning with linear discriminant analysis
Source: Front Pediatr. 2024 Feb 14;12:1328209. doi: 10.3389/fped.2024.1328209 (PMC10899433; doi:10.3389/fped.2024.1328209)
Supplement: Supplementary file 5 [file Table5.docx]

**Supplement Material.5**

**The list of parameters of six hyperparameter-tuned ML models when AHI≥5 were the classification criteria.**

| **the binary classification threshold** | **the type of ML models** | **the parameter of these hyperparameter-tuned ML models (the serial number of 5 repeation when using 5 different random seed value for data splitting)** | | | | |
| --- | --- | --- | --- | --- | --- | --- |
|  |  | **1** | **2** | **3** | **4** | **5** |
|  |  | random seed=786 | random seed=256 | random seed=321 | random seed=423 | random seed=521 |
| an AHI of 5 events/h | LR | C=1.164, class_weight={}, dual=False, fit_intercept=True, intercept_scaling=1, l1_ratio=None, max_iter=1000, penalty='l2', solver='lbfgs', tol=0.0001 | C=0.212 class_weight={}, dual=False, fit_intercept=True, intercept_scaling=1, l1_ratio=None, max_iter=1000, penalty='l2', solver='lbfgs', tol=0.0001 | C=0.493,  class_weight=balanced, dual=False, fit_intercept=True, intercept_scaling=1, l1_ratio=None, max_iter=1000, penalty='l2', solver='lbfgs', tol=0.0001 | C=3.086,  class_weight=balanced, dual=False, fit_intercept=True, intercept_scaling=1, l1_ratio=None, max_iter=1000, penalty='l2', solver='lbfgs', tol=0.0001 | C=0.214,  class_weight=balanced, dual=False, fit_intercept=True, intercept_scaling=1, l1_ratio=None, max_iter=1000, penalty='l2', solver='lbfgs', tol=0.0001 |
|  | LDA | n_components=None,  priors=None,  shrinkage=0.4,  solver=lsqr,  store_covariance=False,  tol=0.0001 | n_components=None,  priors=None,  shrinkage=0.5,  solver=lsqr,  store_covariance=False,  tol=0.0001 | n_components=None,  priors=None,  shrinkage=0.3,  solver=eigen,  store_covariance=False,  tol=0.0001 | n_components=None,  priors=None,  shrinkage=0.4,  solver=lsqr,  store_covariance=False,  tol=0.0001 | n_components=None,  priors=None,  shrinkage=0.5,  solver=lsqr,  store_covariance=False,  tol=0.0001 |
|  | RBF-SVM | C=11.63, break_ties=False, cache_size=200, class_weight='balanced',  coef0=0.0, decision_function_shape='ovr', degree=3, gamma='auto',  kernel='rbf', max_iter=-1, probability=True, shrinking=True, tol=0.001 | C=3.17, break_ties=False, cache_size=200, class_weight='balanced',  coef0=0.0, decision_function_shape='ovr', degree=3, gamma='auto',  kernel='rbf', max_iter=-1, probability=True, shrinking=True, tol=0.001 | C=4.92, break_ties=False, cache_size=200, class_weight='balanced',  coef0=0.0, decision_function_shape='ovr', degree=3, gamma='auto',  kernel='rbf', max_iter=-1, probability=True, shrinking=True, tol=0.001 | C=0.35, break_ties=False, cache_size=200, class_weight='balanced',  coef0=0.0, decision_function_shape='ovr', degree=3, gamma='auto',  kernel='rbf', max_iter=-1, probability=True, shrinking=True, tol=0.001 | C=2.13, break_ties=False, cache_size=200, class_weight='balanced',  coef0=0.0, decision_function_shape='ovr', degree=3, gamma='auto',  kernel='rbf', max_iter=-1, probability=True, shrinking=True, tol=0.001 |
|  | Catboost | nan_mode=Min,  eval_metric=Logloss,  Iterations=260,  sampling_frequency=PerTree,  leaf_estimation_method=Newton,  grow_policy=SymmetricTree,  penalties_coefficient=1,  boosting_type=Plain,  model_shrink_mode=Constant,  feature_border_type=GreedyLogSum,  bayesian_matrix_reg=0.10000000149011612,  l2_leaf_reg=5,  random_strength=0.800000011920929,  rsm=1,  boost_from_average=False,  model_size_reg=0.5,  pool_metainfo_options={'tags': {}},  Subsample=0.800000011920929,  use_best_model=False,  Depth=7,  posterior_sampling=False,  border_count=254,  classes_count=0,  auto_class_weights=None,  sparse_features_conflict_fraction=0,  leaf_estimation_backtracking=AnyImprovement,  best_model_min_trees=1,  model_shrink_rate=0,  min_data_in_leaf=1,  loss_function=Logloss,  learning_rate=0.009999999776482582,  score_function=  Cosine,  leaf_estimation_iterations=10,  bootstrap_type=MVS,  max_leaves=128 | nan_mode=Min,  eval_metric=Logloss,  Iterations=60,  sampling_frequency=PerTree,  leaf_estimation_method=Newton,  grow_policy=SymmetricTree,  penalties_coefficient=1,  boosting_type=Plain,  model_shrink_mode=Constant,  feature_border_type=GreedyLogSum,  bayesian_matrix_reg=0.10000000149011612,  l2_leaf_reg=1,  random_strength=0.800000011920929,  rsm=1,  boost_from_average=False,  model_size_reg=0.5,  pool_metainfo_options={'tags': {}},  Subsample=0.800000011920929,  use_best_model=False,  Depth=2,  posterior_sampling=False,  border_count=254,  classes_count=0,  auto_class_weights=None,  sparse_features_conflict_fraction=0,  leaf_estimation_backtracking=AnyImprovement,  best_model_min_trees=1,  model_shrink_rate=0,  min_data_in_leaf=1,  loss_function=Logloss,  learning_rate=0.00.10000000149011612,  score_function=  Cosine,  leaf_estimation_iterations=10,  bootstrap_type=MVS,  max_leaves=4 | nan_mode=Min,  eval_metric=Logloss,  Iterations=30,  sampling_frequency=PerTree,  leaf_estimation_method=Newton,  grow_policy=SymmetricTree,  penalties_coefficient=1,  boosting_type=Plain,  model_shrink_mode=Constant,  feature_border_type=GreedyLogSum,  bayesian_matrix_reg=0.10000000149011612,  l2_leaf_reg=8,  random_strength=0.800000011920929,  rsm=1,  boost_from_average=False,  model_size_reg=0.5,  pool_metainfo_options={'tags': {}},  Subsample=0.800000011920929,  use_best_model=False,  Depth=3,  posterior_sampling=False,  border_count=254,  classes_count=0,  auto_class_weights=None,  sparse_features_conflict_fraction=0,  leaf_estimation_backtracking=AnyImprovement,  best_model_min_trees=1,  model_shrink_rate=0,  min_data_in_leaf=1,  loss_function=Logloss,  learning_rate=0.00.20000000298023224,  score_function=  Cosine,  leaf_estimation_iterations=10,  bootstrap_type=MVS,  max_leaves=8 | nan_mode=Min,  eval_metric=Logloss,  Iterations=260,  sampling_frequency=PerTree,  leaf_estimation_method=Newton,  grow_policy=SymmetricTree,  penalties_coefficient=1,  boosting_type=Plain,  model_shrink_mode=Constant,  feature_border_type=GreedyLogSum,  bayesian_matrix_reg=0.10000000149011612,  l2_leaf_reg=3,  random_strength=0.20000000298023224,  rsm=1,  boost_from_average=False,  model_size_reg=0.5,  pool_metainfo_options={'tags': {}},  Subsample=0.800000011920929,  use_best_model=False,  Depth=6,  posterior_sampling=False,  border_count=254,  classes_count=0,  auto_class_weights=None,  sparse_features_conflict_fraction=0,  leaf_estimation_backtracking=AnyImprovement,  best_model_min_trees=1,  model_shrink_rate=0,  min_data_in_leaf=1,  loss_function=Logloss,  learning_rate=0.00.004999999888241291,  score_function=  Cosine,  leaf_estimation_iterations=10,  bootstrap_type=MVS,  max_leaves=64 | nan_mode=Min,  eval_metric=Logloss,  Iterations=100,  sampling_frequency=PerTree,  leaf_estimation_method=Newton,  grow_policy=SymmetricTree,  penalties_coefficient=1,  boosting_type=Plain,  model_shrink_mode=Constant,  feature_border_type=GreedyLogSum,  bayesian_matrix_reg=0.10000000149011612,  l2_leaf_reg=100,  random_strength=0,  rsm=1,  boost_from_average=False,  model_size_reg=0.5,  pool_metainfo_options={'tags': {}},  Subsample=0.800000011920929,  use_best_model=False,  Depth=6,  posterior_sampling=False,  border_count=254,  classes_count=0,  auto_class_weights=None,  sparse_features_conflict_fraction=0,  leaf_estimation_backtracking=AnyImprovement,  best_model_min_trees=1,  model_shrink_rate=0,  min_data_in_leaf=1,  loss_function=Logloss,  learning_rate=0.00.05000000074505806,  score_function=  Cosine,  leaf_estimation_iterations=10,  bootstrap_type=MVS,  max_leaves=64 |
|  | AdaBoost | algorithm='SAMME', base_estimator=None, learning_rate=0.5, n_estimators=130, | algorithm='SAMME', base_estimator=None, learning_rate=0.3, n_estimators=30, | algorithm='SAMME', base_estimator=None, learning_rate=0.3, n_estimators=280, | algorithm='SAMME', base_estimator=None, learning_rate=0.15, n_estimators=240, | algorithm='SAMME', base_estimator=None, learning_rate=0.15, n_estimators=120, |
|  | RF | Bootstrap=False,  ccp_alpha=0.0,  class_weight=balanced_subsample,  Criterion=entropy,  max_depth=4,  max_features=sqrt,  max_leaf_nodes=None,  max_samples=None,  min_impurity_decrease=0.0001,  min_impurity_split=None,  min_samples_leaf=6,  min_samples_split=7,  min_weight_fraction_leaf=0.0,  n_estimators=190,  oob_score=False | Bootstrap=True,  ccp_alpha=0.0,  class_weight=balanced,  Criterion=gini,  max_depth=9,  max_features=sqrt,  max_leaf_nodes=None,  max_samples=None,  min_impurity_decrease=0.0005,  min_impurity_split=None,  min_samples_leaf=6,  min_samples_split=9,  min_weight_fraction_leaf=0.0,  n_estimators=290,  oob_score=False | Bootstrap=False,  ccp_alpha=0.0,  class_weight={},  Criterion=gini,  max_depth=3,  max_features=log2,  max_leaf_nodes=None,  max_samples=None,  min_impurity_decrease=0.0005,  min_impurity_split=None,  min_samples_leaf=2,  min_samples_split=2,  min_weight_fraction_leaf=0.0,  n_estimators=210,  oob_score=False | Bootstrap=True,  ccp_alpha=0.0,  class_weight=balanced,  Criterion=gini,  max_depth=9,  max_features=sqrt,  max_leaf_nodes=None,  max_samples=None,  min_impurity_decrease=0.0001,  min_impurity_split=None,  min_samples_leaf=2,  min_samples_split=2,  min_weight_fraction_leaf=0.0,  n_estimators=110,  oob_score=False | Bootstrap=True,  ccp_alpha=0.0,  class_weight=balanced,  Criterion=gini,  max_depth=10,  max_features=sqrt,  max_leaf_nodes=None,  max_samples=None,  min_impurity_decrease=0,  min_impurity_split=None,  min_samples_leaf=4,  min_samples_split=10,  min_weight_fraction_leaf=0.0,  n_estimators=220,  oob_score=False |

**The list of parameters of six hyperparameter-tuned ML models when AHI≥10 were the classification criteria.**

| **the binary classification threshold** | **the type of ML models** | **the parameter of these hyperparameter-tuned ML models (the serial number of 5 repeation when using 5 different random seed value for data splitting)** | | | | |
| --- | --- | --- | --- | --- | --- | --- |
|  |  | **1** | **2** | **3** | **4** | **5** |
|  |  | random seed=786 | random seed=256 | random seed=321 | random seed=423 | random seed=521 |
| an AHI of 10 events/h | LR | C=1.164, class_weight={}, dual=False, fit_intercept=True, intercept_scaling=1, l1_ratio=None, max_iter=1000, penalty='l2', solver='lbfgs', tol=0.0001 | C=0.212 class_weight={}, dual=False, fit_intercept=True, intercept_scaling=1, l1_ratio=None, max_iter=1000, penalty='l2', solver='lbfgs', tol=0.0001 | C=0.493,  class_weight=balanced, dual=False, fit_intercept=True, intercept_scaling=1, l1_ratio=None, max_iter=1000, penalty='l2', solver='lbfgs', tol=0.0001 | C=1.336,  class_weight={}, dual=False, fit_intercept=True, intercept_scaling=1, l1_ratio=None, max_iter=1000, penalty='l2', solver='lbfgs', tol=0.0001 | C=0.28,  class_weight={}, dual=False, fit_intercept=True, intercept_scaling=1, l1_ratio=None, max_iter=1000, penalty='l2', solver='lbfgs', tol=0.0001 |
|  | LDA | n_components=None,  priors=None,  shrinkage=0.4,  solver=lsqr,  store_covariance=False,  tol=0.0001 | n_components=None,  priors=None,  shrinkage=0.4,  solver=eigen,  store_covariance=False,  tol=0.0001 | n_components=None,  priors=None,  shrinkage=0.3,  solver=eigen,  store_covariance=False,  tol=0.0001 | n_components=None,  priors=None,  shrinkage=0.4,  solver=lsqr,  store_covariance=False,  tol=0.0001 | n_components=None,  priors=None,  shrinkage=0.5,  solver=lsqr,  store_covariance=False,  tol=0.0001 |
|  | RBF-SVM | C=21.91, break_ties=False, cache_size=200, class_weight='balanced',  coef0=0.0, decision_function_shape='ovr', degree=3, gamma='auto',  kernel='rbf', max_iter=-1, probability=True, shrinking=True, tol=0.001 | C=3.17, break_ties=False, cache_size=200, class_weight='balanced',  coef0=0.0, decision_function_shape='ovr', degree=3, gamma='auto',  kernel='rbf', max_iter=-1, probability=True, shrinking=True, tol=0.001 | C=4.92, break_ties=False, cache_size=200, class_weight='balanced',  coef0=0.0, decision_function_shape='ovr', degree=3, gamma='auto',  kernel='rbf', max_iter=-1, probability=True, shrinking=True, tol=0.001 | C=0.35, break_ties=False, cache_size=200, class_weight='balanced',  coef0=0.0, decision_function_shape='ovr', degree=3, gamma='auto',  kernel='rbf', max_iter=-1, probability=True, shrinking=True, tol=0.001 | C=2.13, break_ties=False, cache_size=200, class_weight='balanced',  coef0=0.0, decision_function_shape='ovr', degree=3, gamma='auto',  kernel='rbf', max_iter=-1, probability=True, shrinking=True, tol=0.001 |
|  | Catboost | nan_mode=Min,  eval_metric=Logloss,  Iterations=260,  sampling_frequency=PerTree,  leaf_estimation_method=Newton,  grow_policy=SymmetricTree,  penalties_coefficient=1,  boosting_type=Plain,  model_shrink_mode=Constant,  feature_border_type=GreedyLogSum,  bayesian_matrix_reg=0.10000000149011612,  l2_leaf_reg=5,  random_strength=0.800000011920929,  rsm=1,  boost_from_average=False,  model_size_reg=0.5,  pool_metainfo_options={'tags': {}},  Subsample=0.800000011920929,  use_best_model=False,  Depth=7,  posterior_sampling=False,  border_count=254,  classes_count=0,  auto_class_weights=None,  sparse_features_conflict_fraction=0,  leaf_estimation_backtracking=AnyImprovement,  best_model_min_trees=1,  model_shrink_rate=0,  min_data_in_leaf=1,  loss_function=Logloss,  learning_rate=0.009999999776482582,  score_function=  Cosine,  leaf_estimation_iterations=10,  bootstrap_type=MVS,  max_leaves=128 | nan_mode=Min,  eval_metric=Logloss,  Iterations=60,  sampling_frequency=PerTree,  leaf_estimation_method=Newton,  grow_policy=SymmetricTree,  penalties_coefficient=1,  boosting_type=Plain,  model_shrink_mode=Constant,  feature_border_type=GreedyLogSum,  bayesian_matrix_reg=0.10000000149011612,  l2_leaf_reg=1,  random_strength=0.800000011920929,  rsm=1,  boost_from_average=False,  model_size_reg=0.5,  pool_metainfo_options={'tags': {}},  Subsample=0.800000011920929,  use_best_model=False,  Depth=2,  posterior_sampling=False,  border_count=254,  classes_count=0,  auto_class_weights=None,  sparse_features_conflict_fraction=0,  leaf_estimation_backtracking=AnyImprovement,  best_model_min_trees=1,  model_shrink_rate=0,  min_data_in_leaf=1,  loss_function=Logloss,  learning_rate=0.00.10000000149011612,  score_function=  Cosine,  leaf_estimation_iterations=10,  bootstrap_type=MVS,  max_leaves=4 | nan_mode=Min,  eval_metric=Logloss,  Iterations=20,  sampling_frequency=PerTree,  leaf_estimation_method=Newton,  grow_policy=SymmetricTree,  penalties_coefficient=1,  boosting_type=Plain,  model_shrink_mode=Constant,  feature_border_type=GreedyLogSum,  bayesian_matrix_reg=0.10000000149011612,  l2_leaf_reg=6,  random_strength=0.6000000238418579,  rsm=1,  boost_from_average=False,  model_size_reg=0.5,  pool_metainfo_options={'tags': {}},  Subsample=0.800000011920929,  use_best_model=False,  Depth=3,  posterior_sampling=False,  border_count=254,  classes_count=0,  auto_class_weights=None,  sparse_features_conflict_fraction=0,  leaf_estimation_backtracking=AnyImprovement,  best_model_min_trees=1,  model_shrink_rate=0,  min_data_in_leaf=1,  loss_function=Logloss,  learning_rate=0.00.30000001192092896,  score_function=  Cosine,  leaf_estimation_iterations=10,  bootstrap_type=MVS,  max_leaves=8 | nan_mode=Min,  eval_metric=Logloss,  Iterations=260,  sampling_frequency=PerTree,  leaf_estimation_method=Newton,  grow_policy=SymmetricTree,  penalties_coefficient=1,  boosting_type=Plain,  model_shrink_mode=Constant,  feature_border_type=GreedyLogSum,  bayesian_matrix_reg=0.10000000149011612,  l2_leaf_reg=3,  random_strength=0.20000000298023224,  rsm=1,  boost_from_average=False,  model_size_reg=0.5,  pool_metainfo_options={'tags': {}},  Subsample=0.800000011920929,  use_best_model=False,  Depth=6,  posterior_sampling=False,  border_count=254,  classes_count=0,  auto_class_weights=None,  sparse_features_conflict_fraction=0,  leaf_estimation_backtracking=AnyImprovement,  best_model_min_trees=1,  model_shrink_rate=0,  min_data_in_leaf=1,  loss_function=Logloss,  learning_rate=0.00.004999999888241291,  score_function=  Cosine,  leaf_estimation_iterations=10,  bootstrap_type=MVS,  max_leaves=64 | nan_mode=Min,  eval_metric=Logloss,  Iterations=240,  sampling_frequency=PerTree,  leaf_estimation_method=Newton,  grow_policy=SymmetricTree,  penalties_coefficient=1,  boosting_type=Plain,  model_shrink_mode=Constant,  feature_border_type=GreedyLogSum,  bayesian_matrix_reg=0.10000000149011612,  l2_leaf_reg=5,  random_strength=0.6000000238418579,  rsm=1,  boost_from_average=False,  model_size_reg=0.5,  pool_metainfo_options={'tags': {}},  Subsample=0.800000011920929,  use_best_model=False,  Depth=8,  posterior_sampling=False,  border_count=254,  classes_count=0,  auto_class_weights=None,  sparse_features_conflict_fraction=0,  leaf_estimation_backtracking=AnyImprovement,  best_model_min_trees=1,  model_shrink_rate=0,  min_data_in_leaf=1,  loss_function=Logloss,  learning_rate=0.00.05000000074505806,  score_function=  Cosine,  leaf_estimation_iterations=10,  bootstrap_type=MVS,  max_leaves=256 |
|  | AdaBoost | algorithm='SAMME', base_estimator=None, learning_rate=0.5, n_estimators=130, | algorithm='SAMME', base_estimator=None, learning_rate=0.15, n_estimators=90, | algorithm='SAMME', base_estimator=None, learning_rate=0.5, n_estimators=210, | algorithm='SAMME', base_estimator=None, learning_rate=0.5, n_estimators=210, | algorithm='SAMME', base_estimator=None, learning_rate=0.5, n_estimators=60, |
|  | RF | Bootstrap=True,  ccp_alpha=0.0,  class_weight=balanced,  Criterion=gini,  max_depth=10,  max_features=sqrt,  max_leaf_nodes=None,  max_samples=None,  min_impurity_decrease=0,  min_impurity_split=None,  min_samples_leaf=6,  min_samples_split=7,  min_weight_fraction_leaf=0.0,  n_estimators=190,  oob_score=False | Bootstrap=True,  ccp_alpha=0.0,  class_weight=balanced,  Criterion=gini,  max_depth=9,  max_features=sqrt,  max_leaf_nodes=None,  max_samples=None,  min_impurity_decrease=0.0005,  min_impurity_split=None,  min_samples_leaf=6,  min_samples_split=9,  min_weight_fraction_leaf=0.0,  n_estimators=290,  oob_score=False | Bootstrap=True,  ccp_alpha=0.0,  class_weight=balanced,  Criterion=gini,  max_depth=3,  max_features=sqrt,  max_leaf_nodes=None,  max_samples=None,  min_impurity_decrease=0.0005,  min_impurity_split=None,  min_samples_leaf=2,  min_samples_split=2,  min_weight_fraction_leaf=0.0,  n_estimators=210,  oob_score=False | Bootstrap=True,  ccp_alpha=0.0,  class_weight=balanced,  Criterion=gini,  max_depth=9,  max_features=sqrt,  max_leaf_nodes=None,  max_samples=None,  min_impurity_decrease=0.0001,  min_impurity_split=None,  min_samples_leaf=2,  min_samples_split=2,  min_weight_fraction_leaf=0.0,  n_estimators=110,  oob_score=False | Bootstrap=True,  ccp_alpha=0.0,  class_weight=balanced,  Criterion=gini,  max_depth=10,  max_features=sqrt,  max_leaf_nodes=None,  max_samples=None,  min_impurity_decrease=0,  min_impurity_split=None,  min_samples_leaf=4,  min_samples_split=10,  min_weight_fraction_leaf=0.0,  n_estimators=220,  oob_score=False |
